# miR156 switches on vegetative phase change under the regulation of redox signals in apple seedlings

**DOI:** 10.1038/s41598-017-14671-8

**Published:** 2017-10-27

**Authors:** Xiao Lin Jia, Ya Kun Chen, Xiao Zhao Xu, Fei Shen, Qing Bo Zheng, Zhen Du, Yi Wang, Ting Wu, Xue Feng Xu, Zhen Hai Han, Xin Zhong Zhang

**Affiliations:** Institute for Horticultural Plants, China Agricultural University, No. 2 Yuanmingyuan West Rd, Beijing, 100193 China

## Abstract

In higher plants, miR156 regulates the vegetative phase change via the target *SBP/SPL* genes. The regulation of miR156 during ontogenetic processes is not fully understood. In the apple genome, of 31 putative *MdMIR156* genes that encode pre-miR156, seven were dominantly expressed. However, the transcript levels of only *MdMIR156a5* and *MdMIR156a12* decreased significantly during the vegetative phase change, which was consistent with the mature miR156 level, indicating that miR156 is under transcriptional regulation. Leaf H_2_O_2_ content was higher in the adult phase than in the juvenile phase because of excess H_2_O_2_ accumulation in chloroplasts. When *in vitro* shoots were treated with menadione, diphenyleneiodonium, L-2-oxothiazolidine-4-carboxylic acid or buthionine sulphoximine, the expressions of *MdMIR156a5*, *MdMIR156a12*, and as well miR156 were coordinated with reduced glutathione (GSH) contents and glutathione/glutathione disulfide ratio but not H_2_O_2_ contents. Alteration of miR156 expression level by *MdMIR156a6-*overexpressing or miR156-mimetic transgenic *Nicotiana benthamiana* did not cause a corresponding change in reactive oxygen species or GSH status. Collectively, the results indicate that the vegetative phase change in apple is controlled by the *MdMIR156a5* and *MdMIR156a12* transcriptional regulatory network in response to the plastid–nucleus redox signals, such as GSH.

## Introduction

Perennial plants undergo several developmental transitions during the life cycle, including the transition from an embryonic to post-embryonic mode of growth, the vegetative phase change (juvenile-to-adult vegetative transition), and the floral transition (vegetative-to-reproductive transition)^1,2^. In apple seedlings, the vegetative phase change occurs at approximately the 80th node, and natural floral induction is initiated at about the 120th node^3^. The vegetative phase change usually occurs once in a life cycle, but this process is reversible (defined as rejuvenation) under certain circumstances, under which the shoot meristem regains juvenility and most juvenile traits after reproductive maturity is attained^4–6^. The vegetative phase change is targeted by plant breeders to shorten the breeding cycle, whereas rejuvenation is critical for plant nurseries and propagators of many perennial woody plants to achieve rapid vegetative growth, to improve rooting ability and to realize efficient proliferation^7^.

The microRNA miR156 regulates the vegetative phase change via inhibition of the target *SQUAMOSA-PROMOTER BINDING PROTEIN-LIKE (SPL)* genes^8,9^. In *Malus* species, during phase change, miR156 expression declines at about the 80th node and attains a minimum level at about the 140th node; on the other hand, during rejuvenation, miR156 levels are recovered in rejuvenated *in vitro* shoots after several subculture cycles^6,10^. The miRNAs can be overexpressed in transformants driven by 35 S promoter or otherwise can be successfully knocked-down by transgenic plants expressing a miRNA-resistant version of a miRNA target or modifying the sequence of the *AtIPS1* gene^11–15^. Overexpression of miR156 in transgenic *Populus × canadensis* reduces the transcripts of miR156-targeted *SPL* genes and drastically prolongs the juvenile phase^16^. The expressions of *NbSPLs* decreased or increased at least five-fold changes in 35 S:*MdMIR156a6* or MIM156 transgenic tobacco (*Nicotiana benthamiana*) lines, respectively^17^.

MicroRNAs can be regulated at transcription, post-transcriptional cleavage or both stages. The processing of miRNAs from long primary transcripts (pri-miRNAs) requires the activity of several proteins, including DICER-LIKE 1 (DCL1), the double stranded RNA-binding protein HYPONASTIC LEAVES 1 (HYL1), and the zinc-finger protein SERRATE (SE)^18–23^. Loss-of-function mutants of *HYL1*, *SE* or *ARGONAUTE 1 (AGO1*) cause defects in the timing of the juvenile phase in *Arabidopsis* ^24–28^.

The ontogenetic signal upstream of miR156 is proposed to originate from leaf primordia. Defoliation disrupts miR156 expression, which implies that a mobile signal(s) is (are) derived from the pre-existing leaves^29,30^. One potential mobile signal that triggers the juvenile-to-adult transition is sugar. Recent study reveals that trehalose-6-phosphate (T6P) pathway regulates flowering at two sites in plant. In the leaves, TREHALOSE-6-PHOSPHATE SYNTHASE 1 (TPS1) activity is required for the induction of the florigen FT, even under inductive photoperiod^31^. In addition, the T6P pathway affects the expression of important flowering-time and flower-patterning genes at the shoot apical meristem independently of the photoperiod pathway^31,32^. The loss of TPS1 causes *Arabidopsis thaliana* to flower extremely late^33,34^. Sucrose inhibits pri-miR156 transcription and processing. Supplying *Arabidopsis* plants with exogenous sugar reduces the levels of *MIR156A* and *MIR156C* transcripts by 40%, whereas sugar deprivation increases their expression^35,36^. However, the *Arabidopsis* mutant *gin2-1*, which lacks HEXOKINASE 1 (HXK1), is only slightly precocious in the transition to the adult phase, and thus sugar may not be the only factor that regulates miR156 expression^35^.

Redox signaling hub interacting with phytohormone signaling network controls growth, differentiation, development and environmental response in plants^37^. Redox homeostasis is well maintained by the precisely regulated generation and scavenging of H_2_O_2_, superoxide (O_2_^−^), singlet oxygen (^**1**^O_2_) and other types of reactive oxygen species (ROS)^38^. H_2_O_2_, the most abundant ROS in plants, is produced mainly in chloroplast, apoplast and mitochondria and H_2_O_2_ itself is an inorganic second messenger acts on many redox sensitive regulation processes^39,40,41^. Under none stressful conditions, the dominant H_2_O_2_ production sites in plant are chloroplast and peroxisome, and chloroplastic H_2_O_2_ induces early signal responses^42^.

Glutathione (GSH) and ascorbate (ASC) coupling is a central hub of redox regulation, and GSH is considered as an important redox buffer in plants^43,44^. GSH concentration, GSH/glutathione disulfide (GSSG) ratio and the cellular/subcellular compartmentation are tightly regulated^45,46,47^.

Several changes in ROS generation and scavenging were age related in some organisms. In *Drosophila melanogaster*, *Musca domestica* and *Mus musculus*, production of GSH declines and that of ROS increases with age^48,49^. In apple seedlings, the expression of miR156 decreased gradually during ontogenesis. H_2_O_2_ contents, glutathione reductase activity and the expressions of some *MdGR* gene family members increased remarkably. However, the GSH content and GSH to GSSG ratio declined^10,50^. When H_2_O_2_ concentrations of *in vitro* shoots of an apple seedling are manipulated with menadione (MED) or diphenyleneiodonium (DPI) treatment, the concentrations of GSH was observed extremely lower in MED treated *in vitro* shoots than in untreated control, and the relative expression of miR156 decreased to a significantly low level. In DPI treated *in vitro* shoots, a slight but statistically significant increase in GSH was detected, a drastic increase in miR156 expression was observed. When GSH contents were altered with L-2-oxothiazolidine-4-carboxylic acid (OTC) or buthionine sulphoximine (BSO) application, miR156 expression varies concomitantly, but no substantial changes in H_2_O_2_ concentrations were detected in OTC and BSO treated *in vitro* shoots compared to the control^10^. It seems that the H_2_O_2_ levels are not a direct factor than GSH affecting miR156 expression, but the exact relationship between ROS, GSH and miR156 remains unclear.

Cellular ROS can be formed in almost all kinds of organelles. In non-photosynthetic organisms and mammalian cells, more than 90% of cellular ROS is produced in the mitochondria as a result of untimely spontaneous transfer of electrons to oxygen, primarily from complexes I and III of the respiratory chain^51,52^. In animals, age-related ROS accumulate in mitochondria and the endoplasmic reticulum^52^. Recent studies have identified respiratory burst oxidase homologues (*Rboh*), plant homologues of the catalytic subunit of phagocyte NADPH oxidase (gp91phox), as a source of ROS during the apoplastic oxidative burst^53^. In green plants, excitation of pigments and photosynthetic electron transfer reactions in an oxygen-rich environment lead to the production of ROS^54^. In higher plants, the site of the dramatically higher H_2_O_2_ content in the reproductive phase compared with the juvenile phase is presently unknown.

To understand whether the phase change-associated decrease in miR156 expression level is due to transcriptional regulation by redox homeostasis in plants, in this study on apple (*Malus domestica*) we first confirmed the ontogenesis-related members of the *MdMIR156* gene family. The response of *MdMIR156* transcription to the changes in redox status was evaluated using *in vitro* shoots, and the site of the phase change-associated ROS generation was investigated by transmission electron microscopy.

## Results

### miR156 is under transcriptional regulation during phase change

**Figure 1:**
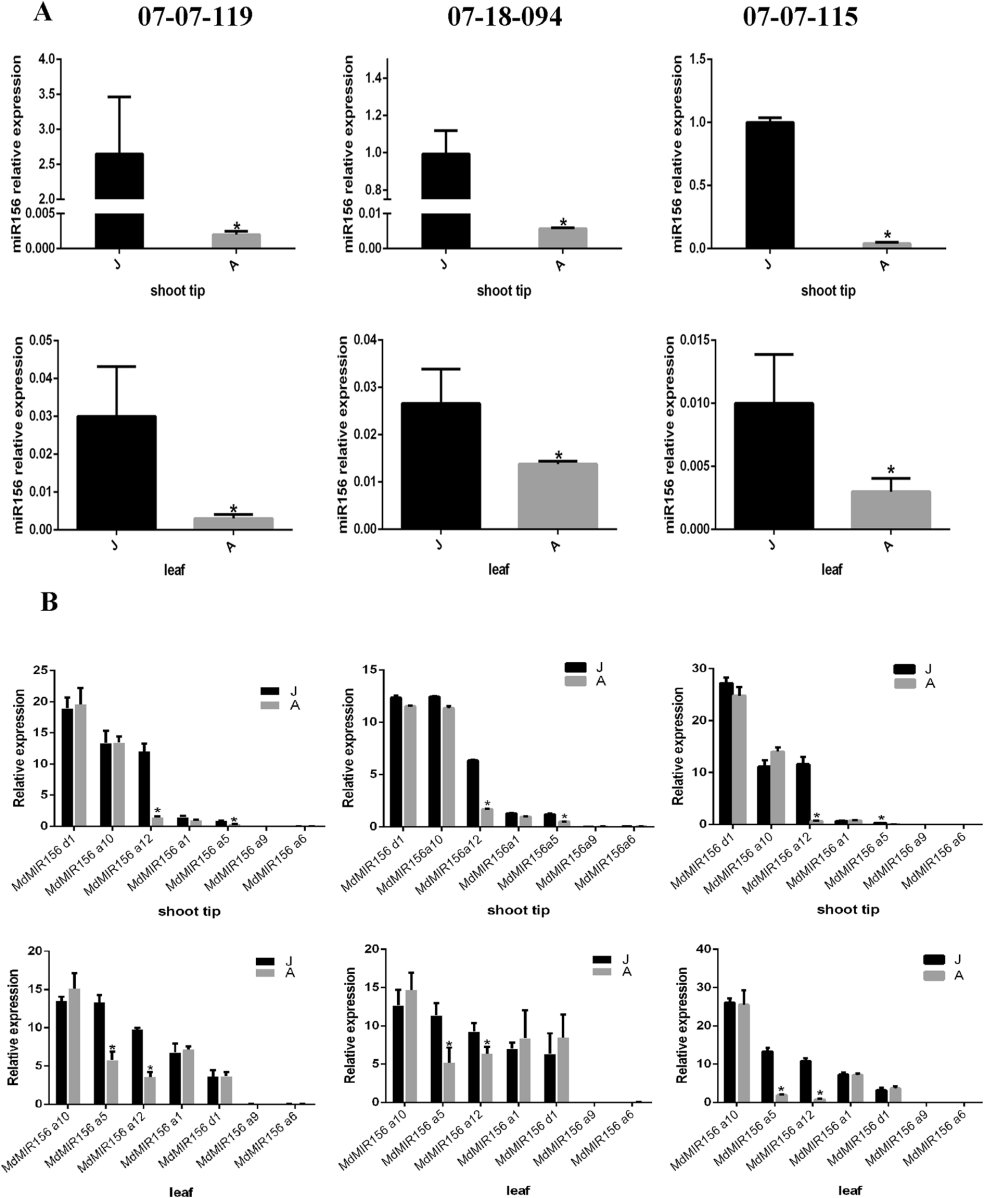
Quantitative expression of miR156 (**A**) and major members of the *MdMIR156* gene family (**B**) in shoot tips and leaves in the juvenile (J) and adult (**A**) phase of three individuals of *Malus asiatica* ‘Zisai Pearl’ × *M. domestica* ‘Red Fuji’. Error bars represent the SD of three experimental replicates. Asterisk represents *p* < 0.05 (Duncan’s multiple-range test).

In both leaf and shoot tip samples, the expression level of mature miR156 in the adult phase was dramatically lower than that in the juvenile phase (Fig. 1A).

Twenty-seven gene family members containing SPL-box were identified in the apple genome, 14 of which were putative targets of miR156 (*MdSPL3*, 4, 6, 7, 10, 11, 12, 18, 20, 21, 22, 23, 24, 26). *MdSPL6* did not express and the transcripts of *MdSPL10&11* or *MdSPL21&22* were identical to each other. Six *MdSPL* members (*MdSPL26*, 24, 3, 20, 18 and 23) were dominantly expressed in the three hybrids (Fig. S1). The reduced levels of miR156 during phase change were also concomitant with significantly and robustly increased expressions of its targets, *MdSPL26*, *MdSPL23*, and *MdSPL10&11* (Fig. S1).

**Figure 2:**
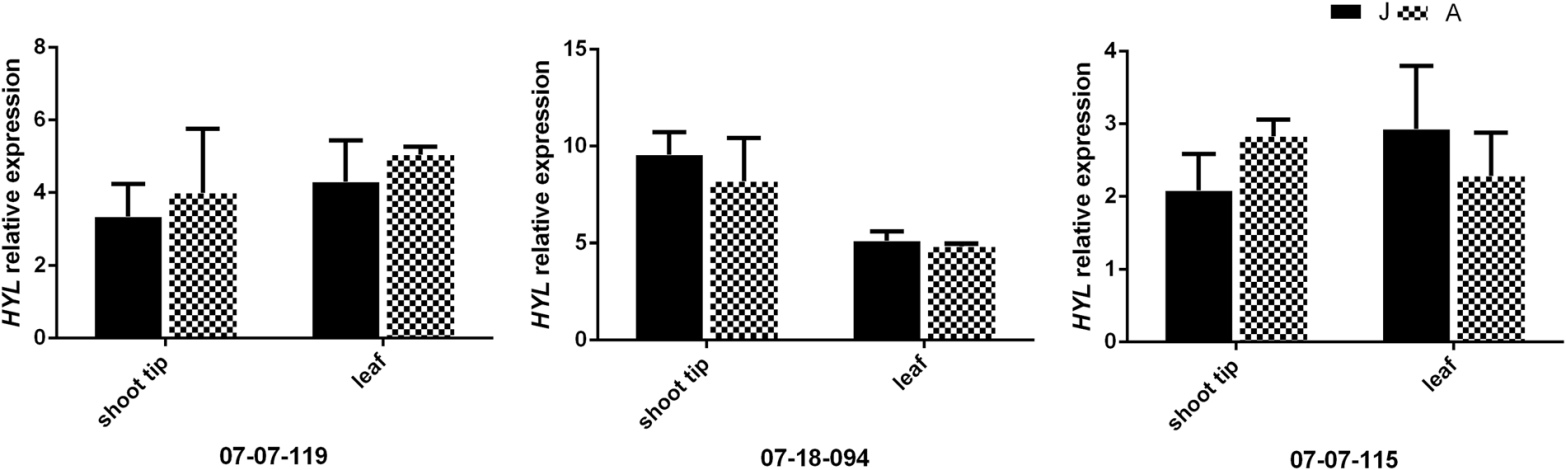
Quantitative expression of *MdHYL1* in shoot tips and leaves in juvenile (J) and adult phase (**A**) of three individuals of *Malus asiatica* ‘Zisai Pearl’ × *M. domestica* ‘Red Fuji’. Error bars represent the SD of three experimental replicates.

In the apple genome, there are 31 putative *MdMIR156* genes encode pre-miR156 (Fig. S5, Table S7). Of these 31 genes, seven were dominantly expressed in shoot tip tissue and the leaf (*MdMIR156d1*, *a10*, *a12*, *a1*, *a5*, *a9* and *a6*). Despite the transcripts of *MdMIR156d1, MdMIR156a10* and *MdMIR156a1* were slightly higher at adult phase than juvenile phase, but their differences were not statistically significant. The transcripts of *MdMIR156a5* and *MdMIR156a12* exhibited significantly lower levels in the adult phase than in the juvenile phase, which was closely correlated with mature miR156 expression (Fig. 1B). No notable changes in the expression of *MdHYL1*, which encodes the double-stranded RNA-binding protein HYL1, were detected between the juvenile and adult phases in either shoot tip or leaf samples (Fig. 2). These data indicate that miR156 expression is, at least partially, under transcriptional regulation, and that *MdMIR156a5* and *MdMIR156a12* are key family members responsive to ontogenetic cues.

**Figure 3:**
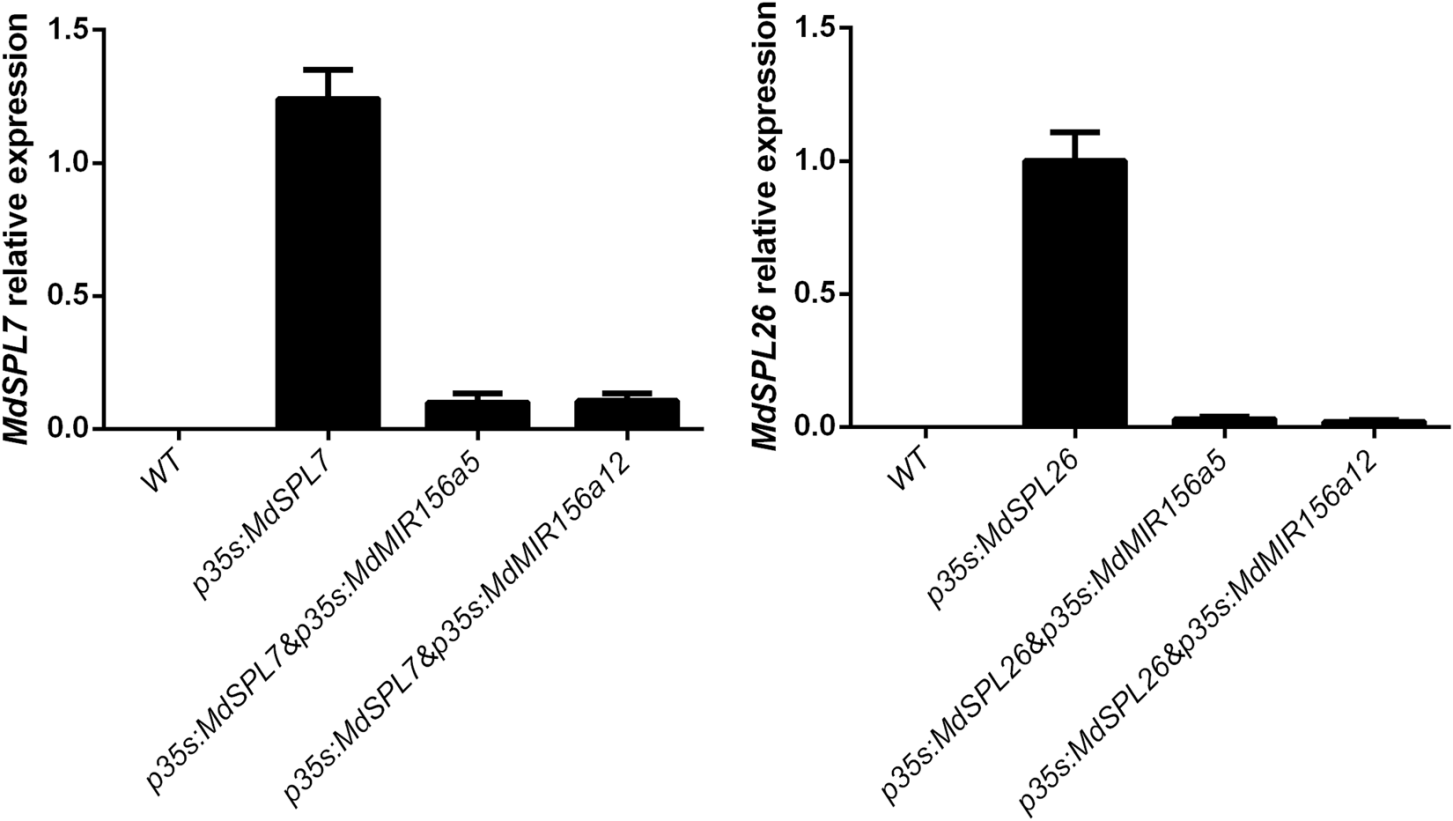
Quantitative expression of *MdSPL7* and *MdSPL26* in wild and transgenic lines of *Nicotiana benthamiana*. The expression constructs *MdMIR156* (*a5* or *a12*) and *MdSPL* (*MdSPL7* or *MdSPL26*) were mixed and transformed simultaneously into *N. benthamiana* by injection. The experiment was performed with three replicates injected with five plaques. The pBI121 vector harboring the *GUS* reporter gene was also injected to confirm the conversion efficiency. *GUS* was used as the reference gene. Error bars represent the SD of three experimental replicates.

In transiently transformed tobacco, quantitative RT-PCR analysis showed that *MdSPL7* and *MdSPL26* transcript levels were reduced significantly in both *MdMIR156a5* and *MdMIR156a12* co-transformed lines (Fig. 3). These results indicated that transcripts of *MdMIR156a5* and *MdMIR156a12* may degrade *MdSPLs*, the target of miR156, and thus are indicated to be precursor genes of mature miR156.

### miR156 is regulated downstream of GSH and ROS

Given that *in vitro* shoots derived from adult-phase explants undergo rejuvenation, the juvenility of the *in vitro* shoot changes with subculture cycles^6^. In the preliminary experiments, in suspension cells derived from apple ‘*Orin*’ leaves, mature miR156 expression level and redox parameters remained constant and robust with successive subculture cycles. Therefore, suspension cells were an ideal system to use for redox treatments (Figs S2–S4).

**Figure 4:**
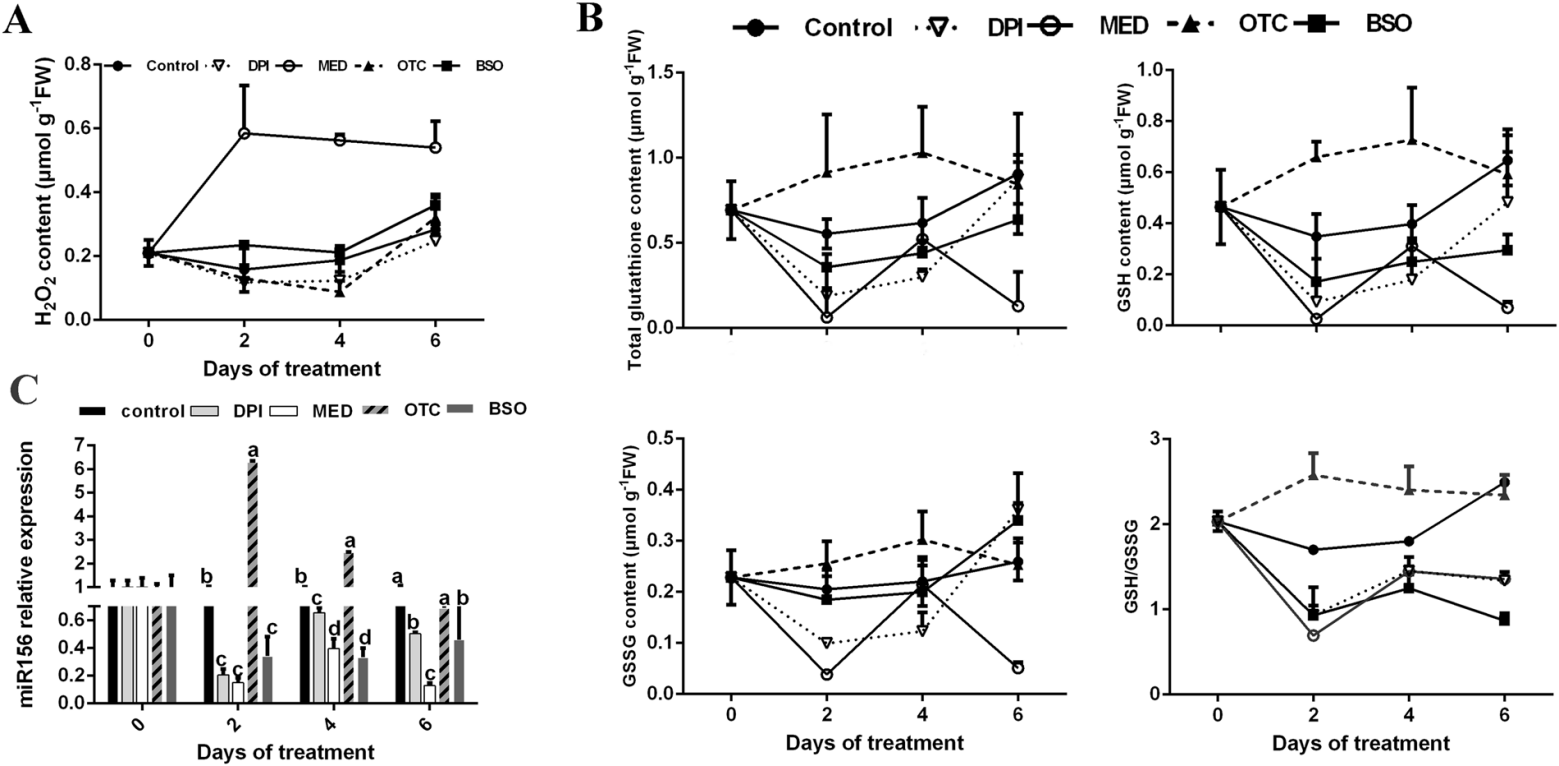
Changes in hydrogen peroxide (H_2_O_2_) concentration (**A**), glutathione (GSH) content, glutathione/glutathione disulfide (GSH/GSSG) (**B**) and relative expression of mature miR156 (**C**) of suspension cells of apple ‘*Orin*’ leaf treated with redox-modulating chemicals. The suspension cells were cultured for six days in medium supplemented with either 50 μM menadione (MED), 5 μM diphenyleneiodonium (DPI), 50 μM L-2-oxothiazolidine-4-carboxylic acid (OTC) or 0.5 mM buthionine sulphoximine (BSO). Error bars represent the SD of three biological replicates.

The H_2_O_2_ concentration of suspension cells on MED-supplemented medium increased significantly as expected on days 2–6 of treatment (Fig. 4A). In contrast, H_2_O_2_ concentration in DPI-treated suspension cells was lower than that of the untreated control on days 2–4 (Fig. 4A). No significant changes in H_2_O_2_ were detected in response to OTC and BSO treatment compared with the untreated control (Fig. 4A).

The concentrations of GSH, GSSG, and GSH + GSSG, and the GSH/GSSG ratio decreased significantly on days 2–6 in the MED treatment (Fig. 4B). Compared with the untreated control, in the DPI treatment no obvious changes in GSSG concentrations occurred throughout the experimental period, whereas a significant decline in GSH, GSH + GSSG and GSH/GSSG ratio were detected on days 2–6 (Fig. 4B). On days 2–4 of OTC treatment, a dramatic increase in concentration of reduced GSH was observed and consequently the GSH/GSSG ratio was elevated (Fig. 4B). In the suspension cells cultured in BSO-supplemented medium, significant depletions in reduced GSH and total GSH + GSSG concentrations were observed on days 2–6 of treatment. However, the GSSG concentration did not change, resulting in a low GSH/GSSG ratio throughout the experiment (Fig. 4B).

The relative expression level of miR156 in the MED, DPI, and BSO treatments were significantly decreased on days 2–6, whereas a dramatic increase in miR156 expression level was observed in the OTC treatment except on day 6 (Fig. 4C). These results prompted us to examine the expressions of miR156 targets *MdSPL26*, *MdSPL23*, and *MdSPL10&11*, which responded to phase change. qRT-PCR analysis revealed that the expressions of *MdSPL26* was elevated in BSO treatment but decreased in response to OTC treatment (Fig. S6). Thus the impacts of GSH on miR156 expression were established.

**Figure 5:**
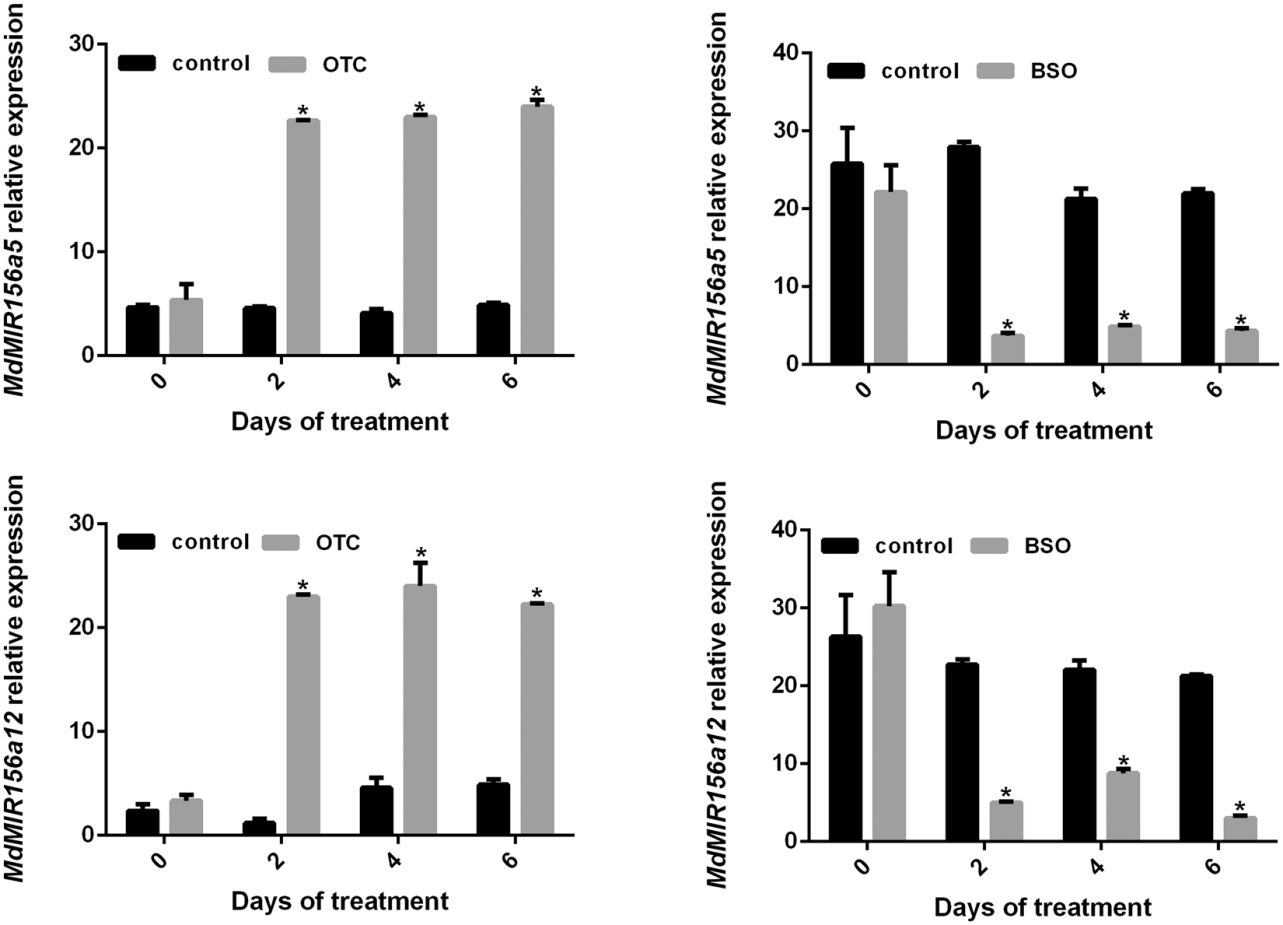
The relative expression of *MdMIR156a5* and *MdMIR156a12* of suspension cells of apple ‘*Orin*’ leaf treated with redox-modulating chemicals. The suspension cells were cultured for six days in medium supplemented with either 50 μM L-2-oxothiazolidine-4-carboxylic acid (OTC) or 0.5 mM buthionine sulphoximine (BSO). Error bars represent the SD of three biological replicates. Asterisk represents *p*<0.05 (Ducan's multiple-range test).

We examined the transcript levels of *MdMIR156a5* and *MdMIR156a12* in response to OTC and BSO treatment. The transcript levels of *MdMIR156a5* and *MdMIR156a12* increased by up to 20-fold in response to OTC treatment, but decreased significantly in response to BSO treatment (Fig. 5). These changes were consistent with the changes in miR156 expression level.

Thus, when suspension cells were treated with exogenous redox modulators, the changes in transcript levels of *MdMIR156a5*, *MdMIR156a12*, and mature miR156 were consistent with the changes in reduced GSH concentration and the GSH/GSSG ratio, and showed no direct correspondence with H_2_O_2_ concentration.

**Figure 6:**
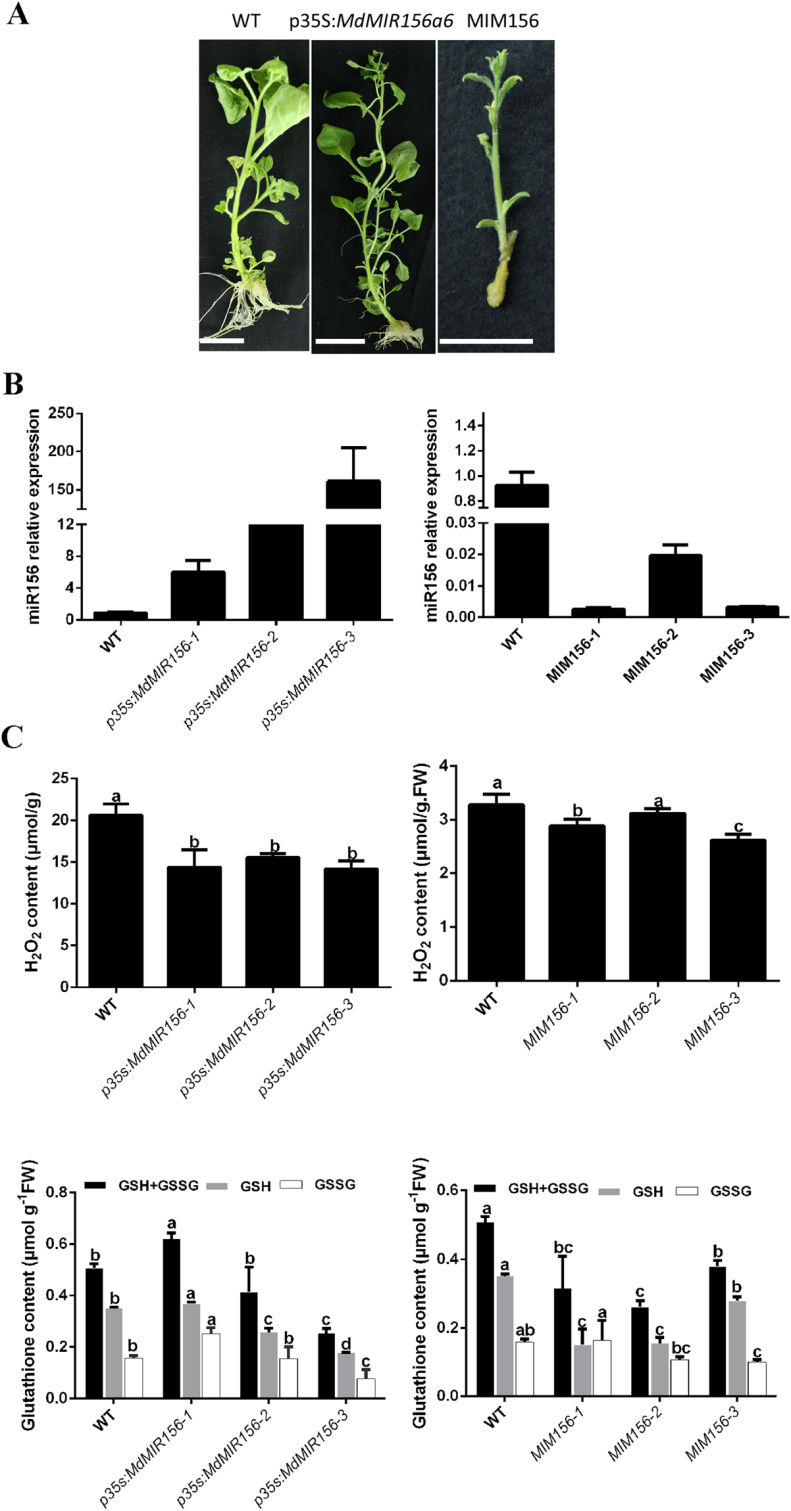
*Nicotiana benthamiana* transformant *in vitro* shoots constitutively expressing MIM156 or *MdMIR156a6*. (**A**) Phenotypes of *Nicotiana benthamiana* transformant. (**B**) Expression analysis of miR156 in *Nicotiana benthamiana* transformant *in vitro* shoots constitutively expressing MIM156 or *MdMIR156a6* compared with the wild type (WT). *5S* was used as a loading control. (**C**) Changes in H_2_O_2_ concentration and glutathione concentration in *Nicotiana benthamiana* transformant *in vitro* shoots constitutively expressing MIM156 or *MdMIR156a6* compared with the wild type (WT). Error bars represent the SD of three biological replicates. Different letters indicate statistical significance (*p* < 0.05; Duncan’s multiple-range test).

To test whether the changes in miR156 expression level may affect redox homeostasis, an expression construct containing the miR156 precursor *MdMIR156a6* under the control of the *Cauliflower mosaic virus* 35 S promoter, and a target mimic, consisting of a non-cleavable RNA that formed a non-productive interaction with a complementary miR156 (MIM156) to inhibit the activity of miR156, were genetically transformed into *Nicotiana benthamiana*. The transgenic plants showed multiple morphological changes; 35 S:*MdMIR156a6*-overexpressing plants showed increased number of leaves (double that of the wild type [WT]), shorter internode length, and inhibition of flowering, whereas the transgenic MIM156-overexpressing plants flowered profusely (Fig. 6A).

Three independent *MdMIR156a6* overexpression transgenic lines (p35S:*MdMIR156-1*, p35S:*MdMIR156-2*, and p35S:*MdMIR156-3*) and three miR156 mimetic transgenic lines (MIM156-1, MIM156-2, and MIM156-3) were chosen for further analysis. *MdMIR156a6* overexpression transgenic plants accumulated much higher quantities of mature miR156 than the WT plants, whereas miR156 expression was greatly inhibited in the MIM156 transgenic plants (Fig. 6B).

The H_2_O_2_ and GSH concentrations showed no regular differences between the *MdMIR156a6-*overexpressing and miR156-mimetic transgenic lines, and also between the WT and transgenic lines (Fig. 6C). These data indicated that the change in miR156 expression level did not affect H_2_O_2_ and glutathione concentrations constantly. Taken together, these results indicated that miR156 was regulated downstream of ROS and GSH.

### Changes in sugar metabolism during phase change

To determine whether sugar is involved in the phase change in apple seedlings, we examined the contents of glucose, fructose, the activity of TPS and the expression of *MdHXK* gene family members. The glucose content was significantly higher in adult phase than those in juvenile phase in 07-07-115, but no significant differences were detected between juvenile and adult tissue in 07-07-119 and 07-18-094, and no significant variation in TPS activity was found between the he juvenile and adult phases (Fig. S7). Of the ten members of *MdHXK* gene in apple genome, eight (except *MdHXK6* and *MdHXK10*) were actively expressed in leaf tissue in apple. To get better convincing data, nine hybrid trees were used for analyzing the variations in *MdHXKs* expression patterns between juvenile and adult phase and to consider if *MdHXKs* were co-expressed with miR156. The data showed obviously that the miR156 expressions were significantly higher in the juvenile phase, which was consistent among all the nine hybrids (Fig. S8). The expressions of *MdHXK5* and *MdHXK8* were higher in juvenile than in adult leaves in 07-07-115, and 07-07-119, respectively, however, the expressions of *MdHXK1*, *MdHXK2* and *MdHXK5* were significantly higher in the adult phase in 07-05-017, 07-18-094, 07-06-140 and 07-07-119, respectively (Fig. S8). Together, no substantial and consistent variations in sugar metabolism can be concluded between the juvenile and the adult phase.

### The site of phase*-*related elevation in ROS concentration

**Figure 7:**
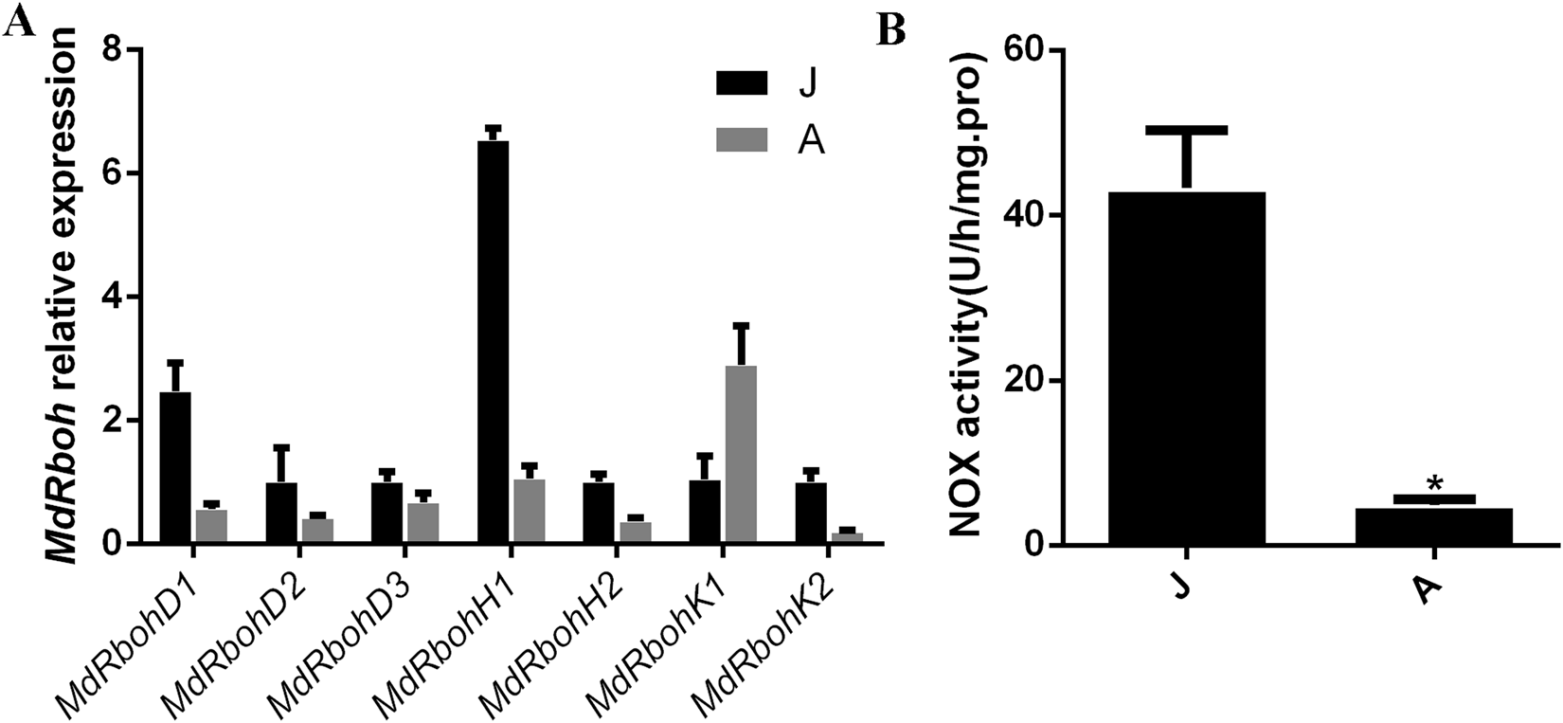
Quantitative expression of members of the *MdRboh* gene family (**A**) and NADPH oxidase (NOX) activity (**B**) in the juvenile (J) and adult (**A**) phase of *Malus asiatica* ‘Zisai Pearl’ × *M. domestica* ‘Red Fuji’. Error bars represent the SD of three biological replicates. Asterisk represents *p* < 0.05 (Duncan’s multiple-range test).

To confirm whether or not the phase-related changes in ROS concentration are generated from the apoplast, NADPH oxidase (NOX) activity and expression of *Rboh* gene family members in leaf samples of apple hybrid trees was measured. The apple genome contains seven members in *MdRboh* gene family, of which six (*D1*, *D2*, *D3*, *H1*, *H2*, and *K2*) showed more active transcription in the juvenile phase, especially *MdRboh H1* (Fig. 7A). Only the transcript level of *MdRboh K1* was higher in the adult phase than in the juvenile phase (Fig. 7A). Consistently, NOX activity was significantly higher in the juvenile phase than in the adult phase (Fig. 7B). Changes in NOX activity and *MdRboh* gene transcription were not consistent with H_2_O_2_ dynamics during the phase change and therefore the ontogenetic-specific elevation in ROS concentration was not generated in the apoplast.

Subsequently we measured the concentration of H_2_O_2_, GSH miR156 and miR156-targeted *AtSPL* genes (*AtSPL*3 and *AtSPL*9) expression in *Atrbohd* and *Atrbohf* mutant. In *Atrbohd* mutant, the concentration of H_2_O_2_, GSH, expressions of miR156 and *AtSPLs* did not differ significantly from the wild-type, in *Atrbohf* mutant, however, there was a significant decrease of GSH content, GSH/GSSG ratio and miR156 transcripts, the expression of *AtSPL9* correspondingly increased (Figs S9, S10). These data from *Arabidopsis* further support that GSH, but not H_2_O_2_, affects miR156 expression.

**Figure 8:**
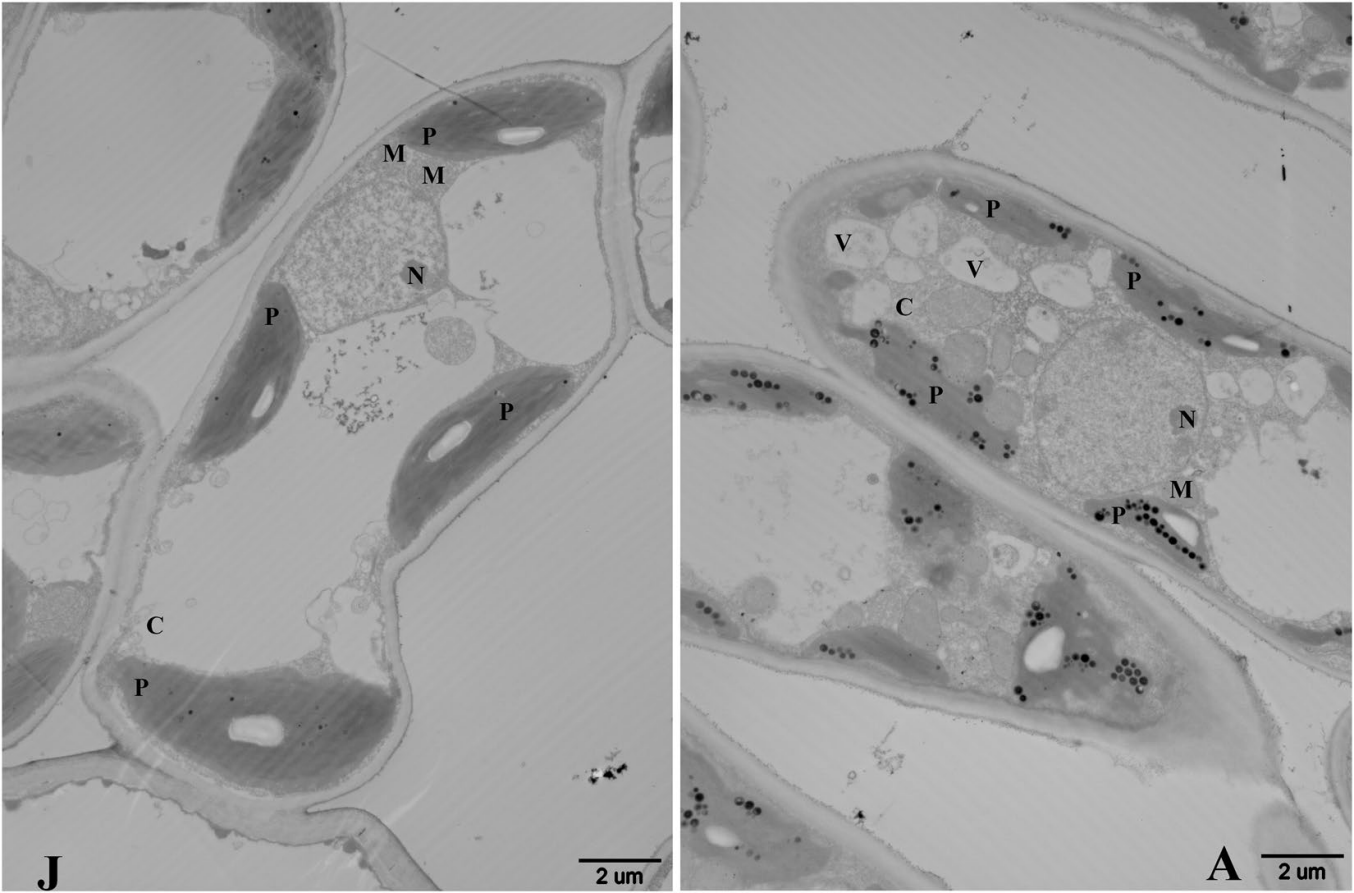
Cytochemical detection of H_2_O_2_ accumulation in the juvenile (left) and adult phase (right) of *Malus asiatica* ‘Zisai Pearl’ × *M. domestica* ‘Red Fuji’. H_2_O_2_ was visualized by transmission electron microscopy at the subcellular level using CeCl_3_ staining. P: plastid, M: mitochondrion, N: nucleus, V: vacuole, C: cytoplasm.

To further confirm if the phase-related ROS are produced in plastids, subcellular H_2_O_2_ compartmentation was visualized using CeCl_3_ staining and transmission electron microscopy. Accumulation of a large amount of H_2_O_2_ was clearly observed in the chloroplast in leaf samples of the adult phase, in contrast, deposits of CeCl_3_ were barely detected in leaf samples of the juvenile phase (Fig. 8). No obvious differences in CeCl_3_ deposition were observed in the cytosol, nucleus, and other subcellular compartments between leaf samples of the juvenile and adult phases. These data indicated that the phase-related ROS are generated and accumulated in plastids in cells of the adult phase.

## Discussion

The microRNA 156 control of the vegetative phase change in plants is under transcriptional regulation. In the apple genome, 31 *MdMIR156* genes were identified by RNA library sequencing and bioinformatic analysis^55^. Prior to the current study, only one *MdMIR156* precursor (*MdMIR156h*) has been experimentally verified to be processed into mature miR156^56^. Then, we have previously validated that *MdMIR156a6* is one of the coding genes for miR156 precursor, because several *NbSPLs* were down-regulated in 35 S:*MdMIR156a6* transgenic tobacco lines, but increased in 35 S:MIM156 lines^17^. We confirmed in this study *MdMIR156a5* and *MdMIR156a12* can also be transcribed and processed into mature miR156 by using the same approaches.

The present results showed that of the seven family members predominantly expressed in leaf and shoot tip tissues, only transcript abundance of *MdMIR156a5* and *MdMIR156a12* were significantly higher in juvenile than adult phases, which is consistent with the dynamics of miR156 expression. In leaf or stem samples of some seedlings, the expressions of *MdMIR156a1*, *MdMIR156a10* and *MdMIR156d1* were slightly higher in adult phase, but the variations were not statistically significant and were inconsistent among seedling individuals. Given that not all the members of the *MdMIR156* gene family responded to ontogenetic signals, additional functions of miR156 may be unexplored. In *Arabidopsis thaliana*, for example, auxin induces expression of *MIR156B* and *MIR156D* in roots and subsequently increases miR156 expression during lateral-root growth^57^. When the H_2_O_2_ or GSH concentrations of suspension cells were altered by BSO or OTC treatment, the levels of *MdMIR156a5* and *MdMIRa12* transcripts as well as the mature miR156 expression level changed correspondingly. Consequently, the changes in miR156 levels were concomitant with the expression of the dominant ontogenesis responsive miR156 target, *MdSPL26*, which is highly consistent with our previous findings in *M. xiaojinensis* ^17^.

The present results are strongly supported by an analogous observation that the developmental stage associated decline in miR156 expression level is partially mediated by sugar at the transcriptional level of *MIR156A* and *MIR156C* in *Arabidopsis* ^35^. *MdHYL1*, a homolog of *HYL1* in *Arabidopsis thaliana* involved in processing of primary miRNA precursors, did not vary in transcription level between the juvenile and adult phases in leaf and shoot tip tissue. This finding is insufficient to exclude the contribution of post-transcriptional processing of primary miR156 precursors on the decline in mature miR156, but it does not conflict with the hypothesized transcriptional regulation of miR156 during the vegetative phase change.

*MdMIR156* transcription is regulated downstream of redox signals. In our previous experiments, in which *in vitro* shoots were treated with redox-modulating chemicals, miR156 transcript levels varied with GSH concentration and GSH/GSSG ratio but not with H_2_O_2_ concentration^10^. In the present study, we used suspension cells to avoid variation in miR156 expression with successive subculture cycles. In response to OTC or BSO treatment, the transcription of *MdMIR156a5*, *MdMIR156a12* and thus mature miR156 varied depending on GSH concentration and GSH/GSSG ratio. Conversely, whereas miR156 transcript level was modulated by transformation with MIM156 and *MdMIR156* overexpression, the H_2_O_2_ and GSH concentration in transgenic *N. benthamiana* plants did not change consistently, which suggested that H_2_O_2_ and GSH are involved upstream of the miR156 transcriptional regulatory network.

The adult-phase-specific ROS are generated in plastids. During the adult phase, the H_2_O_2_ concentration increases dramatically, as does the concentrations or activities of ROS scavengers^10^. These data indicate that the accumulation of H_2_O_2_ is not because of decreased scavenging capacity, but because of elevation in H_2_O_2_ production. Both NOX activity and *MdRboh* transcript levels were higher (except *MdRboh K1*) in the juvenile phase than in the adult phase, therefore the adult-phase-related H_2_O_2_ is not produced in the apoplast. In *Atrbohd* mutant, the H_2_O_2_ and GSH contents did not change, *RbohD* could not be the crucial member in the redox pathways. In *Atrbohf* mutants, the H_2_O_2_ content did not change at the tissue level, but the apoplast H_2_O_2_ decreased^52^. Because the activity of apoplast γ- glutamyltransferase, which degrades oxidized GSH adducts including GSSG for cysteine recycling, is suppressed when apoplast redox environment gets more reductive, the GSH biosynthesis in chloroplast will be suppressed too due to restricted cysteine availability^58,59,60^. Therefore, both GSH concentration and thus miR156 transcript level declined significantly (Fig. S11).

Because sugar is once proposed as a mobile signal associated with the vegetative phase change in *Arabidopsis* ^35,36^. TPS1 activity is necessary for floral induction^31–34^. To date, there are no data illustrating the relationship among sugar, GSH and miR156 expression. In this study, changes in GSH level and miR156 expression were closely correlated, but we did not find significant and consistent variations in sugar contents and TPS enzyme activity between the juvenile and the adult phase in three apple hybrid trees. In further, none of the eight *MdHXK* gene family members exhibited significant and robust variation between the juvenile and the adult phase. We thus also suspected that the variations in *MdHXKs* expressions between ontogenetic phases were caused by the factors independent with miR156 levels, which agreed to the postulation by Yang and the colleagues^35^.

Recently, in *Arabidopsis*, miR159 has been found to modulate vegetative phase change upstream of miR156 through MYB33^61^. Though the expressions of both *pri-MIR156A* and mature miR156 are much higher during growth in *mir159ab* mutant than in wild type Col-0, but they still decline with days after planting, which indicates an independent mechanism is more powerful on regulating vegetative phase change^61^. Guo and the co-workers proposed that miR159-MYB33 pathway acts on vegetative phase change may be dependent on light^61^. In photosynthetic plants, chloroplast is not only the major subcellular light sensor and receptor, but also one of the major ROS generating sites^39–41,42^. Transmission electron microscopic observations revealed that in the adult phase H_2_O_2_ accumulated only in chloroplasts, indicating that plastids are the main site of phase-related ROS production. No differences in H_2_O_2_ concentration in subcellular compartments other than chloroplasts were detected between the juvenile and adult phases, although H_2_O_2_ is permeable across membranes^62^. The other forms of ROS, such as singlet oxygen or superoxide anion radicals, are considered to be impermeable^63^. Although ROS molecules are established as modulators of gene expression, it is widely accepted that ROS themselves cannot act as signaling molecules in the nucleus because of their high reactivity^64^. Hence, we propose that subsequent retrograde redox signals, rather than chloroplast ROS *per se*, may act on *MdMIR156* transcription. During phase change in apple, the contents of H_2_O_2_ and GSH varied significantly within a considerable extent, in chemically-treated apple *in vitro* shoots, concentrations of H_2_O_2_ and GSH changed drastically, even to an extreme level^10,65,66,67^. But in all cases, miR156 levels varied with GSH concentration and GSH/GSSG ratio but not directly with H_2_O_2_ concentration^10^. In the present experiment, the H_2_O_2_ concentration of suspension cells was either enhanced in response to MED or inhibited by DPI treatment, whereas both GSH concentration and miR156 transcript level declined significantly. MED and DPI alter apoplast H_2_O_2_ production and the apoplast redox state may affect GSH regeneration activity^58,59,60^. On the other hand, GSH levels were altered by OTC or BSO treatment, *MdMIR156s* and mature miR156 transcription varied with GSH concentration, but H_2_O_2_ concentration did not change throughout the experimental period. In *Atrbohf* mutant, the miR156 transcription also changed with GSH concentration. These findings implied that GSH may act downstream of ROS in the *MdMIR156* transcription regulatory network. We propose that GSH may be an element of the plastid-nucleus retrograde redox signals, because GSH nucleus sequestration functions as a retrograde signal closely associated with developmental signals^68,69^. Nuclear GSH may create suitable redox environments for DNA synthesis and repair^70^. As a result, GSH stimulates cell proliferation and cell differentiation in meristematic tissues^71,72^. In future studies on the ontogenesis in plants, we recommend that greater attention should be paid to GSH.

## Conclusions

During the vegetative phase change in apple, ROS accumulate and miR156 transcription declined. The phase-related ROS were generated and accumulated in plastids. In the apple genome, of the 31 putative genes that encode precursors of miR156, *MdMIR156a5* and *MdMIR156a12* responded to ontogenetic regulation, and these two genes were transcriptionally regulated downstream of redox signals such as GSH.

## Methods

### Plant materials and chemical treatments

Eight-year-old trees raised from hybrid seeds of the cross *Malus asiatica* ‘Zisai Pearl’ × *M. domestica* ‘Red Fuji’ were used. ‘Zisai Pearl’ is a Chinese domestic cultivar originating from Hebei. Given the hyper-heterozygosity of the parents and strong segregation among the hybrids, to estimate the genetic variation between individuals, three intact seedlings grown on their own roots, namely 07-07-115, 07-07-119 and 07-18-094, were sampled as biological replicates. To validate the expression patterns of some genes in a larger population, six additional seedlings were chosen randomly (Fig. S7). Young leaves and unlignified shoot tips were sampled from 1-year-old suckers and annual branches. The samples taken from the 1st to the 80th nodes were defined as the juvenile phase, and those from the 120th node to the canopy top as the adult phase^73^. The leaves or the shoot tips collected from the same ontogenetic phase were respectively mixed and divided into three experimental replicates, frozen immediately and stored at −80 °C. The transgenic plants 35 *S:MdMIR156a6* and *35 S:MIM156* were described previously^15^^,^^17^.

To validate the changes in miR156 in response to GSH or redox homeostasis alteration, an ideal experimental system with constant and robust miR156 expression is necessary, because in plant the ROS or GSH levels and miR156 expression varied with the process of vegetative phase change and as well subculture cycles during rejuvenation^6,10^. As porcine granulosa cells were used in several studies in mammalian^74,75^, developmentally de-differentiated suspension cells derived from the leaf of *M. domestica* cultivar ‘*Orin*’ were treated by addition of redox modulating chemicals to the culture medium. In ‘*Orin*’ suspension cells, the redox parameters and the expression of miR156 remained constant and robust with successive subculture cycles (Figs S2–S4). The concentrations used in these experiments were optimized in preliminary tests (Supplementary Tables S1–S4). MED (50 µM) (Sigma-Aldrich, Beijing, China) and 5 µM DPI (Sigma-Aldrich) were used as an inducer and inhibitor of H_2_O_2_, respectively, and 50 µM OTC (Sigma-Aldrich) and 0.5 mM BSO (Sigma-Aldrich) were used as a precursor and inhibitor of GSH biosynthesis, respectively. Suspension cells cultured on medium containing no redox-altering reagent were used as the control. The experiment employed a completely randomized experimental design with three replicates. The sampling time points were on days 0, 2, 4 and 6 of the respective treatments.

The *Atrbohd* and *Atrbohf* mutants in *Arabidopsis thaliana* were obtained from College of Biological Sciences, China Agricultural University. The seeds were surface-sterilized with 2.5% NaClO then sown onto plates containing Murashige and Skoog media containing 3% sucrose and 0.75% agar. The measurements were done using two-week-old seedlings. The qRT-PCR primers for *AtSPL3*, *AtSPL9* and *UBQ10* were referenced to Yang *et al*.^35^.

### Relative expression levels of miR156, *MdHYL1*, *MdMIR156* and *MdSPL* family members

The expression levels of mature miR156 were analyzed using the method described by Du *et al*.^10^. From 500 mg of leaf sample, microRNA was extracted using the RNAiso for Small RNA kit (9753Q, Takara, Dalian, China) following the manufacturer’s instructions. For reverse transcription, 2 μg microRNA extract was diluted in 12 μL diethyl pyrocarbonate (DEPC) water plus 1 μL of dNTPs (10 mMol·L-1, Takara, Dalian, China) and 1 μL stem-loop primer (10 μM). The mixture was incubated at 70 °C for 5 min, and then immediately put on ice for 5 min. Afterwards, 1.25 μL dNTPs (10 mMol·L-1, Takara), 2.5 μL buffer (5 × ) (Takara), 0.6 μL RNasin, and 1 μL M-MLV (5U μL^−1^, Takara) were added to the extracted solution. Reverse transcription was accomplished at 42 °C for 1 h, followed by 70 °C for 10 min. RT-qPCR of miR156 was performed as follows:10 μL miRcute miRNA Premix (2×), 0.4 μL upstream primers, 0.4 μL downstream primers and 2 μL template (cDNA), and DEPC water to 20 μL. Program: 94 °C for 2 min, and 40 cycles of 94 °C for 20 s, and 60 °C for 34 s. RT-qPCR was carried out using the ABI 7500 Real-Time PCR System (Applied Biosystems, Foster City, CA, USA). The primer sequences used for miR156 and 5S rRNA are listed in Supplementary Table S5.

For the *MdMIR156* expression assay, total RNA was extracted using a modified cetyltrimethylammonium bromide method^76^. The sequences of all genes were obtained from Xia *et al*.^55^. The mature sequences of microRNAs are designated with ‘miR’ as prefix in the database, whereas miRNA genes are given names of the form such as ath-MIR166a. Lettered suffixes describe distinct loci expressing all related mature miRNAs^77,78^. The sequences of *MdHYL1* (MDP0000304933) and *MdSPLs* were obtained from the apple genome website (http://genomics.research.iasma.it/) by means of a basic local alignment search tool (BLAST) search. *β-Actin* was used as the reference gene. The primer pairs used are listed in Supplementary Table S6.

### Plasmid construction and genetic transformation

To verify that *MdMIR156* genes can be processed into mature miR156, the expression constructs *MdMIR156* (*a5* or *a12*) and *MdSPL* (*MdSPL7* - MDP0000170630 or *MdSPL26-* MDP0000142582) were mixed and transformed simultaneously into *Nicotiana benthamiana* by injection^79^. The experiment was performed with three replicates injected with five plaques. The pBI121 vector containing the *β-glucuronidase* (*GUS*) reporter gene was also injected to confirm the conversion efficiency. The transient expression of the transgenes was detected by quantitative RT-PCR. *GUS* was used as the reference gene.

### Assay of redox homeostasis

Concentrations of H_2_O_2_ and glutathione were determined using methods described previously^10,80^.

### NOX activity and relative expression of *MdRboh* gene family members

The activity of NOX was measured following the method of Yang *et al*.^81^. The sequences of *Rbohs* were obtained from the apple genome website (http://genomics.research.iasma.it/) by means of a BLAST search. Based on the BLASTP search results for the proteins encoded by the examined genes in the National Center of Biotechnology Information database (http://www.ncbi.nlm.nih.gov/), primers were designed using the Primer Express 5.0 software (AuGCT, Beijing, China). The *Rboh* gene members were named in accordance with Darius *et al*.^82^. The primer pairs used are listed in Supplementary Table S8.

### Analysis of sugar contents, TPS activity and *HXK* gene expression

Leaves samples of juvenile and adult phase of three hybrid trees were used in these experiments as biological replicates. Soluble sugars were extracted and the concentrations of glucose and fructose were separated with a high performance liquid chromatography (Agilent Technologies, CA, USA) and were determined with a refractive index detector (Agilent Technologies, CA, USA) according to the protocols described by Liang *et al*.^83^. The TPS enzyme activity was assayed by using the method of Garg *et al*.^84^. The sequences of *HXK*s were obtained from the apple genome website (http://genomics.research.iasma.it/) by means of a BLAST search. The primer pairs for *HXK* gene members were designed with Primer 5.0 and the sequences are listed in Supplementary Table S9.

### Cytochemical detection of H_2_O_2_

Hydrogen peroxide was visualized by transmission electron microscopy at the subcellular level using CeCl_3_ staining^81^. Tissue samples (2 mM × 2 mM) were excised from the leaves of the juvenile or adult phases, then vacuum infiltrated with freshly prepared 5 mM CeCl_3_ in 50 mM 3-(*N*-morpholino)-propane-sulfonic acid at pH 7.2 for 30 min. The tissue samples were then fixed in 2.5% (v/v) glutaraldehyde in 0.2 M sodium phosphate buffer (PBS), pH 7.2, for 1 h at room temperature and kept overnight at 4 °C. After fixation, the tissue samples were washed twice for 10 min in PBS and dehydrated in a graded acetone series (30, 50, 70, 80, 90 and 100% [v/v]), progressively embedded in increasing concentrations of acetone–resin mixtures, and incubated over two 24 h replacements of pure epoxy resin before polymerization at 60 °C for 48 h. Ultrathin sections were cut, using a diamond knife mounted on nickel grids, and examined without staining with a transmission electron microscope (JEM-1230, JEOL, JAPAN) at an accelerating voltage of 80 kV.

### Statistical analysis

All experimental data were subjected to analysis of variance and Duncan’s multiple-range test. Differences were defined as significant at *p* < 0.05.

### Data Availability

All data generated or analysed during this study are included in this published article and its Supplementary Information files.

## Electronic supplementary material

Supplementary file
