# Supplementary material for: miR156 switches on vegetative phase change under the regulation of redox signals in apple seedlings
Source: Sci Rep. 2017 Oct 27;7:14223. doi: 10.1038/s41598-017-14671-8 (PMC5660156; doi:10.1038/s41598-017-14671-8)
Supplement: Supplementary file 1 — Supplementary file [file 41598_2017_14671_MOESM1_ESM.pdf]

**miR156 switches on vegetative phase change under the regulation of redox signals in apple seedlings**

Xiao Lin Jia, Ya Kun Chen, Xiao Zhao Xu, Fei Shen, Qing Bo Zheng, Zhen Du, Yi Wang, Ting Wu, Xue Feng Xu, Zhen Hai Han, Xin Zhong Zhang*

**SUPPLEMENTARY DATA**

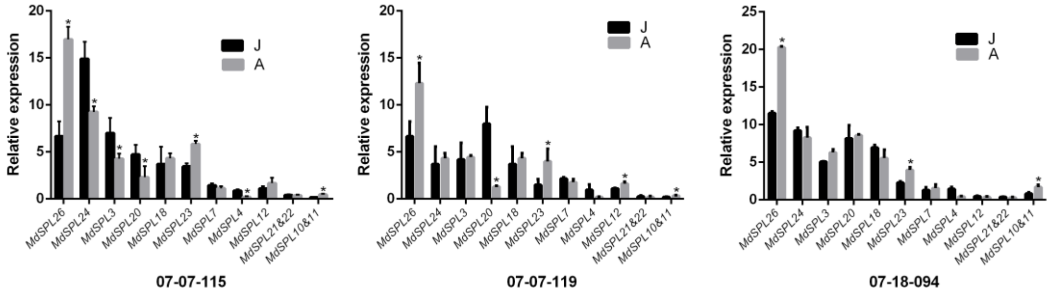


Fig S1. Relative expression of *MdSPLs* in the juvenile (J) and adult (A) phase of three individuals of *Malus asiatica* ‘Zisai Pearl’ × *M. domestica* ‘Red Fuji’. Error bars represent the SD of three biological replicates. Asterisk represents $p < 0.05$ (Duncan’s multiple-range test).

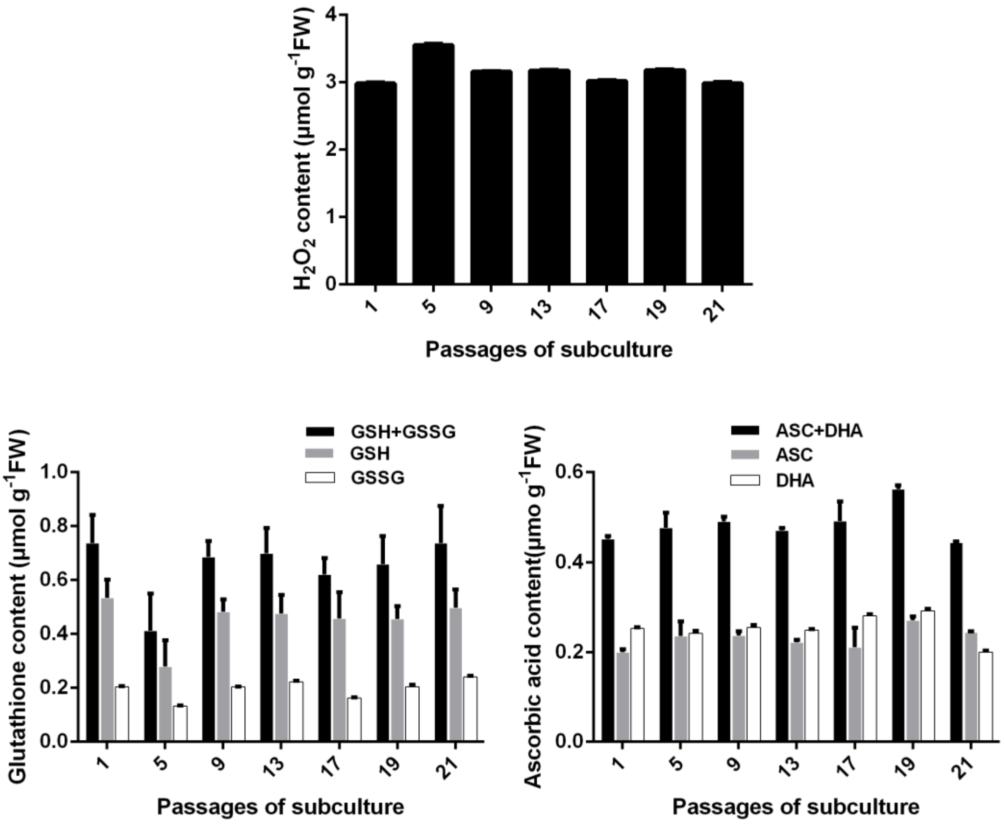


Figure S2. Hydrogen peroxide ($H_2O_2$), glutathione (GSH), glutathione disulfide (GSSG), ascorbic acid (ASC) and dehydroascorbate (DHA) concentrations in suspension cells of apple '*Orin*' leaf during successive subculture cycles.

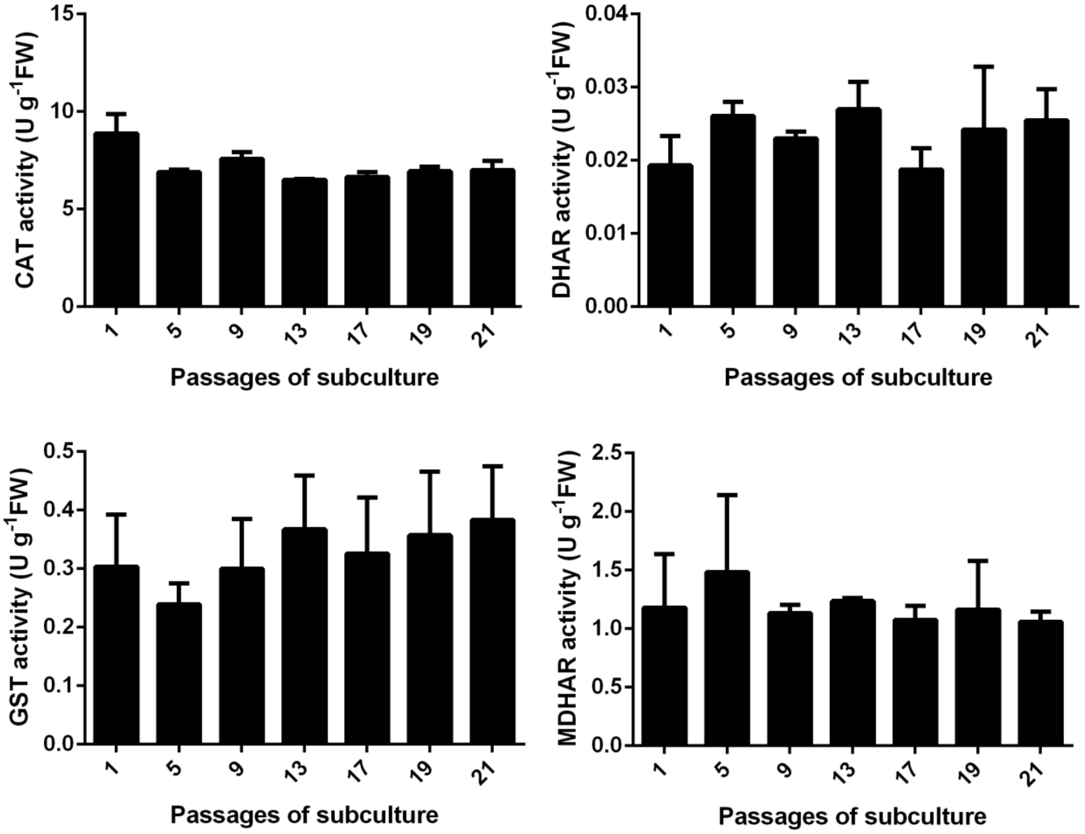


Figure S3. Activity of catalase (CAT), dehydroascorbate reductase (DHAR), glutathione *S*-transferase (GST) and monodehydroascorbate reductase (MDHAR) in suspension cells of apple '*Orin*' leaf during successive subculture cycles.

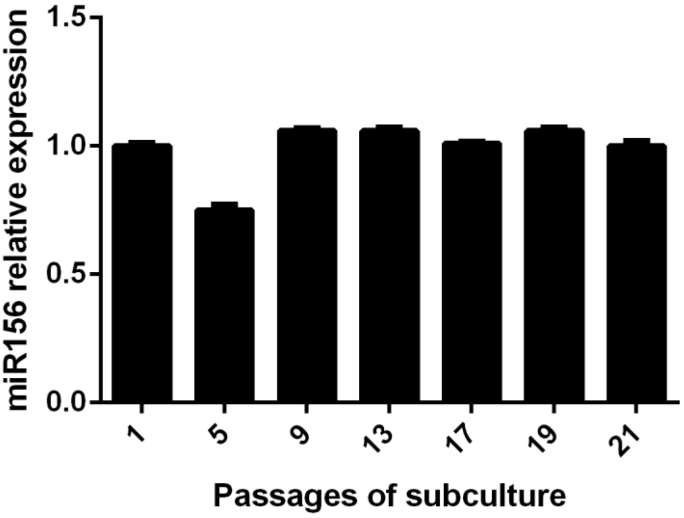


Figure S4. Relative expression of miR156 in suspension cells of apple ‘*Orin*’ leaf during successive subculture cycles.

A

```
MdMIR156a1.seq     .TGACAGAAGAGAGTGAGCAC    20
MdMIR156a2.seq     .TGACAGAAGAGAGTGAGCAC    20
MdMIR156a3.seq     .TGACAGAAGAGAGTGAGCAC    20
MdMIR156a4.seq     .TGACAGAAGAGAGTGAGCAC    20
MdMIR156a5.seq     .TGACAGAAGAGAGTGAGCAC    20
MdMIR156a6.seq     .TGACAGAAGAGAGTGAGCAC    20
MdMIR156a7.seq     .TGACAGAAGAGAGTGAGCAC    20
MdMIR156a8.seq     .TGACAGAAGAGAGTGAGCAC    20
MdMIR156a9.seq     .TGACAGAAGAGAGTGAGCAC    20
MdMIR156a10.seq    .TGACAGAAGAGAGTGAGCAC    20
MdMIR156a11.seq    .TGACAGAAGAGAGTGAGCAC    20
MdMIR156a12.seq    .TGACAGAAGAGAGTGAGCAC    20
MdMIR156a13.seq    .TGACAGAAGAGAGTGAGCAC    20
MdMIR156a14.seq    .TGACAGAAGAGAGTGAGCAC    20
MdMIR156a15.seq    .TGACAGAAGAGAGTGAGCAC    20
MdMIR156b1.seq     CTGACAGAAGATAGAGAGCAC    21
MdMIR156b2.seq     CTGACAGAAGATAGAGAGCAC    21
MdMIR156b3.seq     CTGACAGAAGATAGAGAGCAC    21
MdMIR156b4.seq     CTGACAGAAGATAGAGAGCAC    21
MdMIR156c1.seq     TTGACAGAAGAGAGAGAGCAC    21
MdMIR156c2.seq     TTGACAGAAGAGAGAGAGCAC    21
MdMIR156c3.seq     TTGACAGAAGAGAGAGAGCAC    21
MdMIR156c4.seq     TTGACAGAAGAGAGAGAGCAC    21
MdMIR156d1.seq     .TGACAGAAGATAGAGAGCAC    20
MdMIR156d2.seq     .TGACAGAAGATAGAGAGCAC    20
MdMIR156d3.seq     .TGACAGAAGATAGAGAGCAC    20
MdMIR156d4.seq     .TGACAGAAGATAGAGAGCAC    20
MdMIR156e1.seq     TTGACAGAAGATAGAGAGCAC    21
MdMIR156e2.seq     TTGACAGAAGATAGAGAGCAC    21
MdMIR156f1.seq     .TGACAGAAGAAAGTGAGCAC    20
MdMIR156f2.seq     .TGACAGAAGAAAGTGAGCAC    20
Consensus           tgacagaaga ag gagcac
```

B

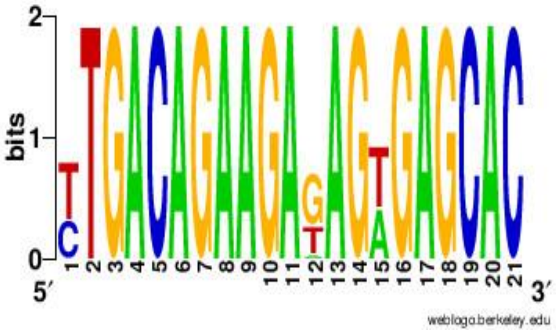


Figure S5. A. Multiple alignment of the MdMIR156s obtained with DNAMAN software. B. Sequence logo of the MdMIR156s. The height of the letter at each position represents the degree of conservation.

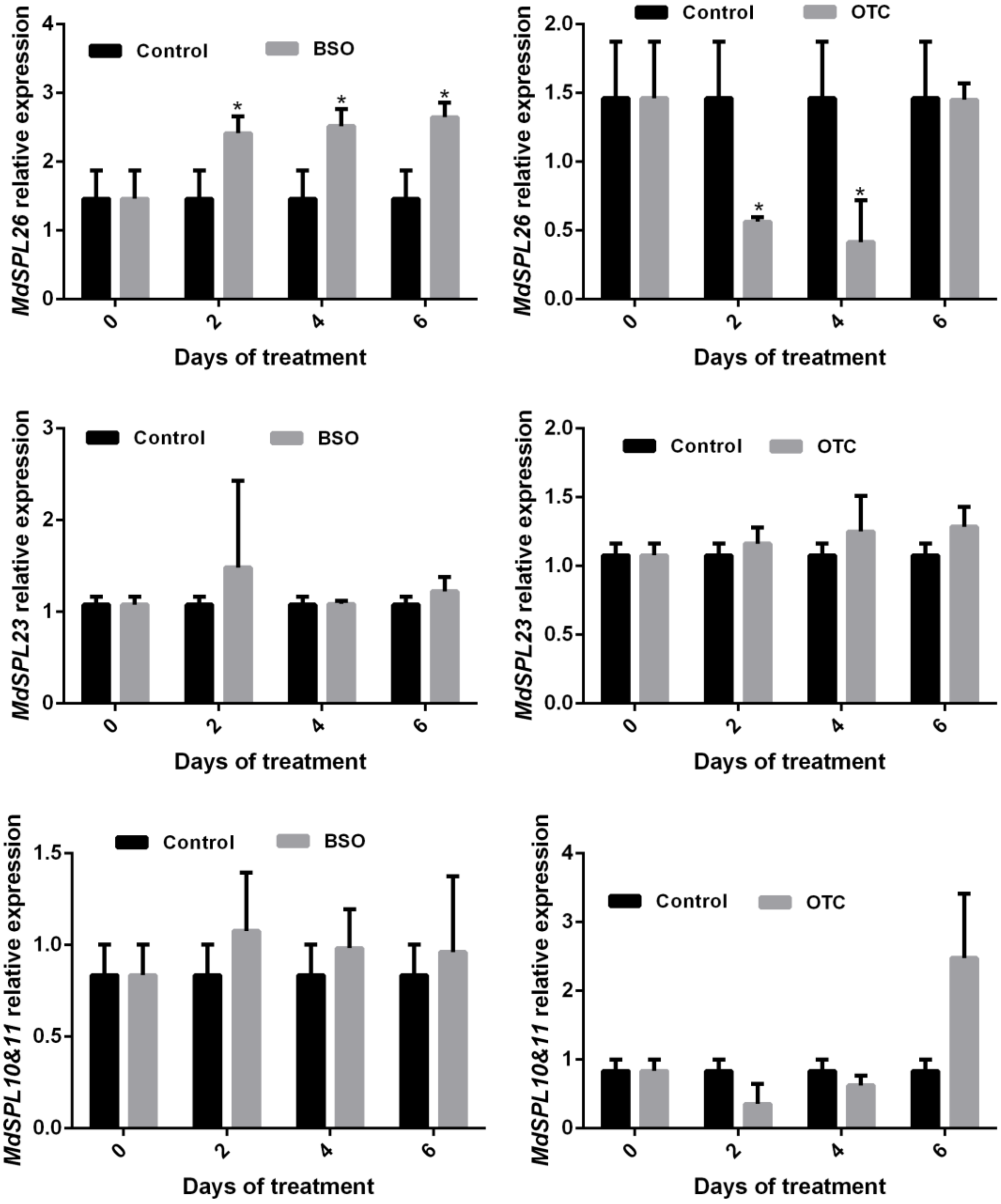


Figure S6. Relative expression of *MdSPL26, MdSPL23* and *MdSPL10&11* of suspension cells of apple '*Orin*' leaf treated with redox-modulating chemicals. The suspension cells were cultured for six days in medium supplemented with either 50 μM L-2-oxothiazolidine-4-carboxylic acid (OTC) or 0.5 mM buthionine sulphoximine (BSO). Error bars represent the SD of three biological replicates.

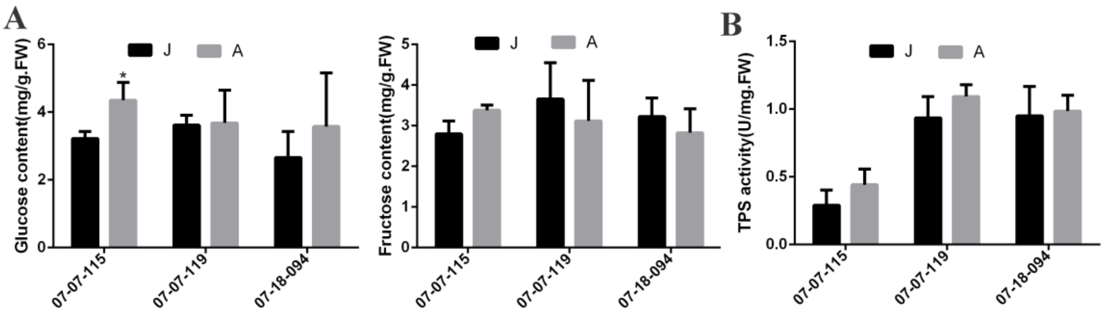


Fig S7. Glucose and Fructose concentrations (A) and activity of Trehalose-6-phosphate synthase (TPS) (B) in the juvenile (J) and adult (A) phase of three individuals of *Malus asiatica* 'Zisai Pearl' × *M. domestica* 'Red Fuji'. Error bars represent the SD of three biological replicates. Asterisk represents $p < 0.05$ (Duncan's multiple-range test).

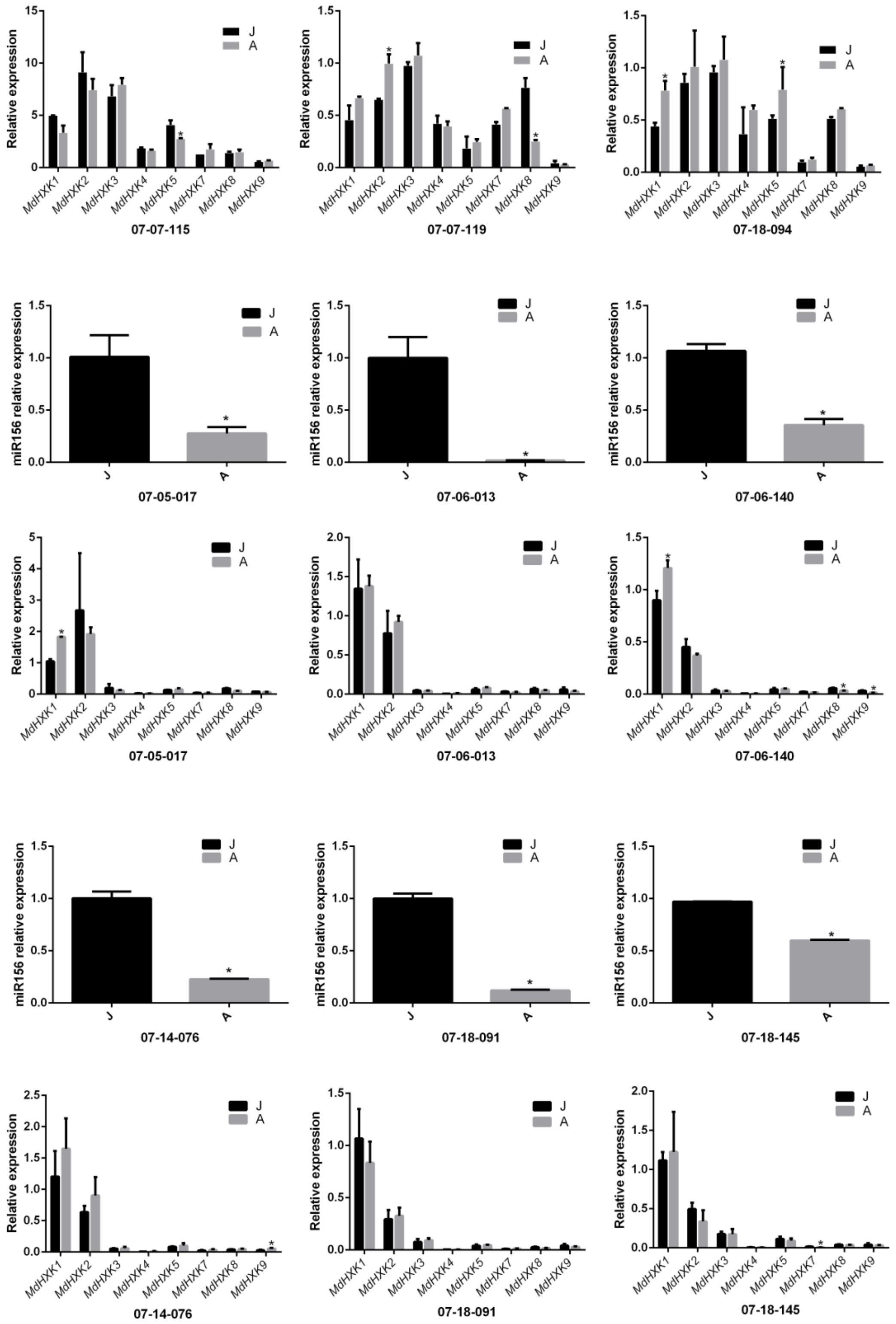


Fig S8. Relative expression of *MdHXKs* in the juvenile (J) and adult (A) phase of three individuals of *Malus asiatica* 'Zisai Pearl' × *M. domestica* 'Red Fuji'. Error bars represent the SD of three biological replicates. Asterisk represents $p < 0.05$ (Duncan's multiple-range test).

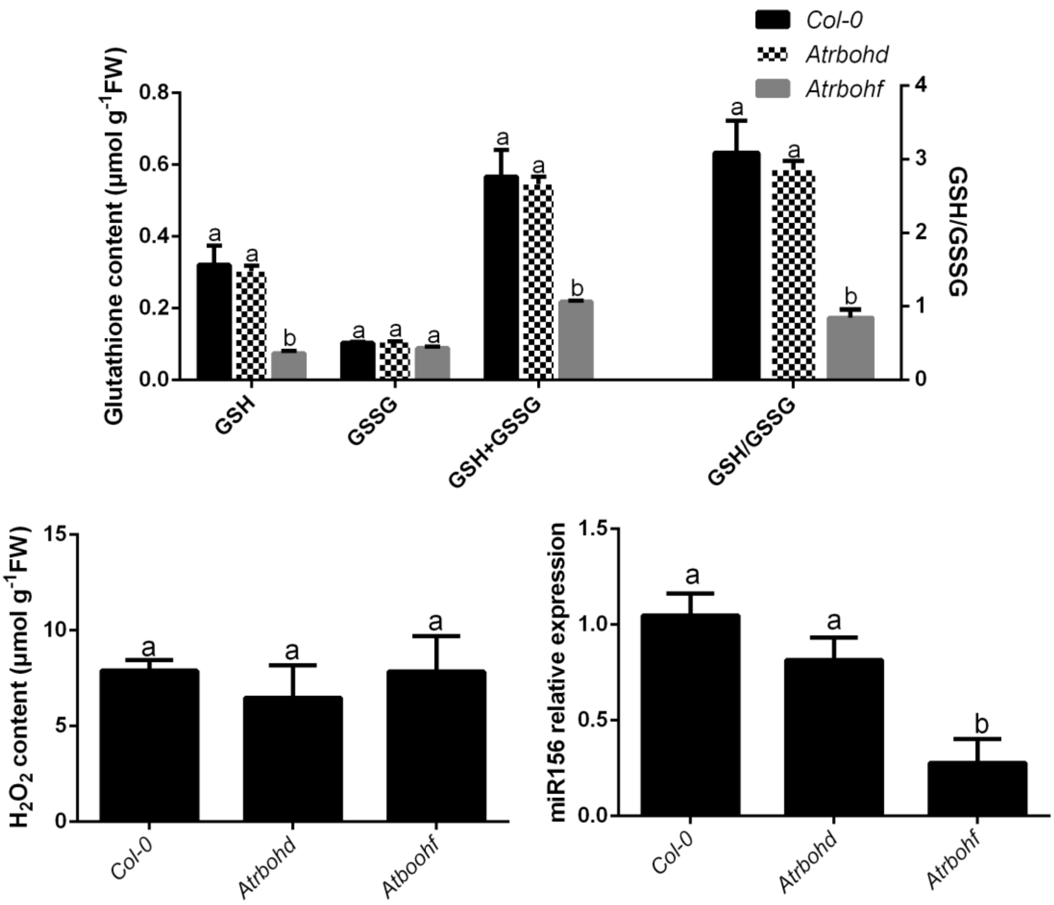


Fig S9. Glutathione (GSH), glutathione disulfide (GSSG) concentrations, GSH/GSSG ratio, Hydrogen peroxide ($H_2O_2$) and miR156 expression in wild type and *rbohd*, *rbohf* mutants of *Arabidopsis thaliana*.

Error bars represent the SD of three biological replicates. Different letters indicate statistical significance ($p <$ 0.05; Duncan's multiple-range test).

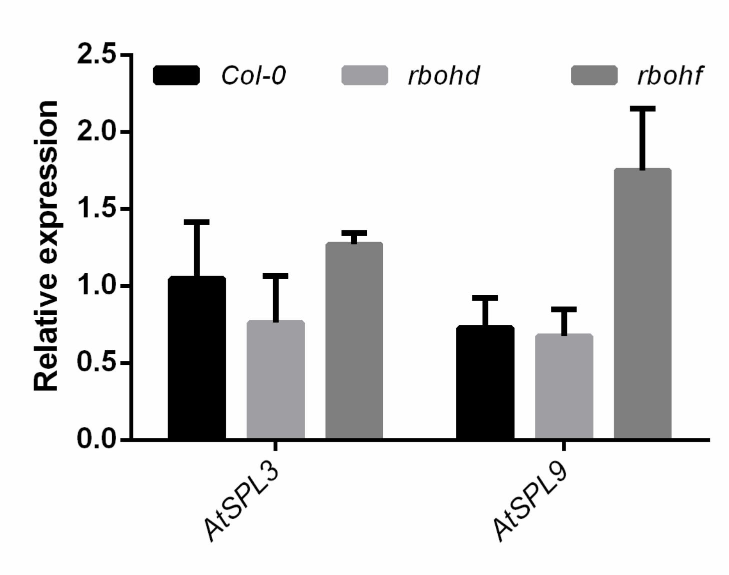


Fig S10. Relative expression of *SPL3* and *SPL9* in wild type and *rbohd*, *rbohf* mutants of *Arabidopsis thaliana*.

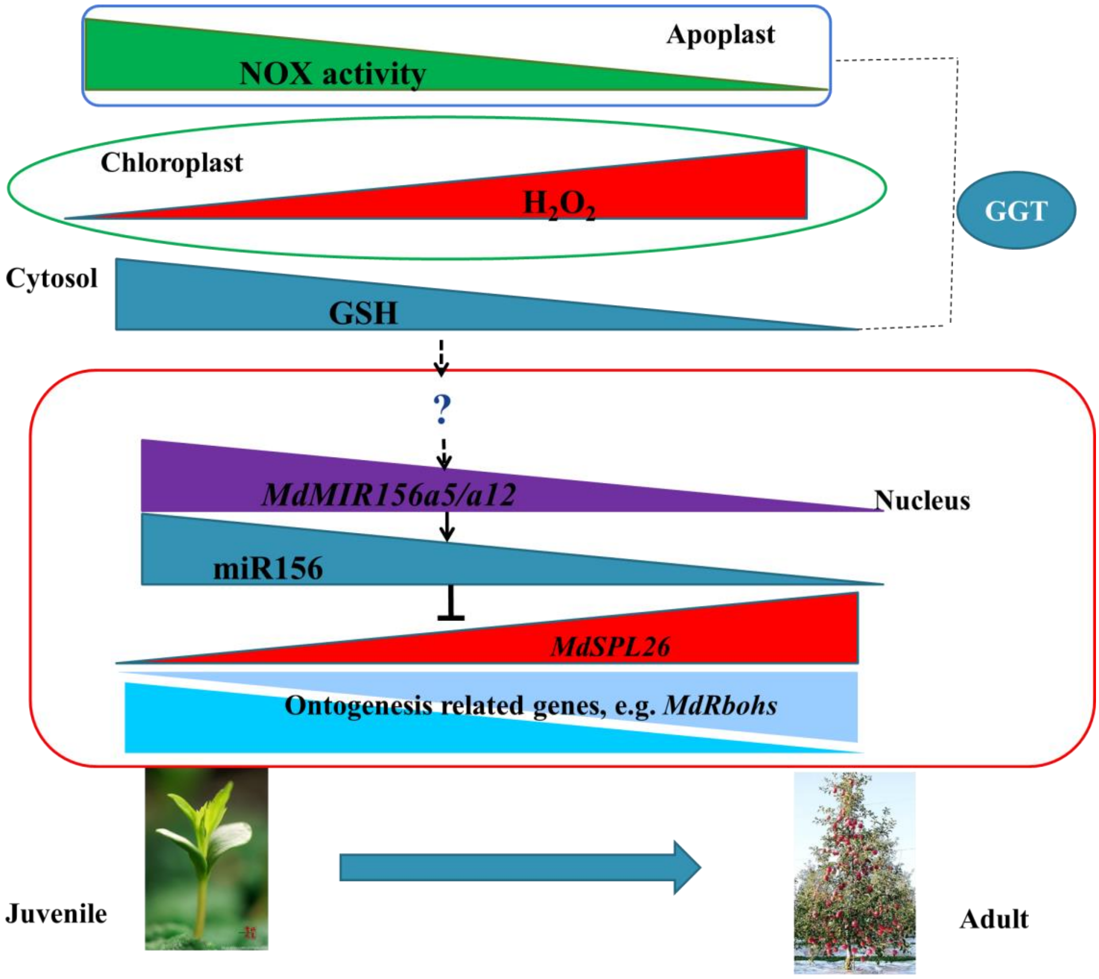


Fig S11. A model for the regulation of miR156 by redox signals to control vegetative phase change in apple seedlings

## 1. The primary transcript sequence of MdMIR156s

The mature miR156 sequences were obtained from miRBase database (http://www.mirbase.org /, Release 21) and the plant microRNA database (PMRB, http:// bioinformatics.cau.edu.cn/PMRD/), then compared with the apple genome (http://genomics.research.iasma.it/) by BLAST. We selected the ~500bp upstream and ~500bp downstream of mature miR156 as the pri-miR156 sequence.

The positions of the mature miR156 embedded on each pri-MdmiR156 member were marked with red colored fonts.

The pri-MdmiR156 transcript, which has been experimentally confirmed to be pri-MdmiR156, was marked with an asterisk following it's name and the corresponding reference (if there is any) is listed just behind the DNA base sequence.

**MdMIR156a1**

GTTTTAAAAGTTATTGTAAGAGGTAGTAATTGCACTTAAATATGAAGGTGAAAAATGTAATTACTCTTTT
TCTTTCCAAAGCACTGGTACTGCTACAAATTGGACACGTAATTGCTAGTGGTGCTAGTAAATTACTTTT
GGTACTTAAATGTCGGGACATGATTCAGATGTGGATGGATCGATGGCAGTCCTTTTATYCACCAACCAG
GCTTTCACCATCAATAATGGAGACCAGAAATCAGGCCCCCTTGCTCAAGTCCTGATGAATTGAAAGATT
YCGCGCGTATACATAGTGAATTATCCTGGTAGATAAGGGCATTCACGGAGCATTCCGAATTCAWAACCC
TAATAAAAACGTGATATGACTCAAGTGAAAGGAAAGCTGCTAGGCTGACCATGGCACTKGGTGGTGTA
ACTAATTAGCTATATATATATRTATATATGTATATTTGTGTATATTGGAAAGCAGAAAGAAACCTGTAGGTT
AAGCTGAGCGTAGTTGACAGAAGAGAGTGAGCACACACAGGCGGYAATTGTATAAAATCATACYTTTG
GGCGGTGCGTGCTCACTTCTCTCTCCGTCAGCTCCTATCTGCGCGCTCGCTGGCTTTATTTCACCACTT
AATTTTGATCGTCAACGTTCAAAACCTTAATTTCTCATCAACGCCCCTTCTCTTCCCGCAAAGGAATGG
GAAATTAAACCAGGCCAGTCCCAGGCTCAGCCCCTTCTACTTTCCCAATTCTCTTTCCAGGTGTTTTTC
TCCCAACCCTATATGCATCTATCTCACATATATGAAACAGATAGCTAGATACCCTCTGATTCCCAGCTCTC
ACTTTTCTTGTTTGGGAAATGGAGAAGTTGTGTATATATTTATAGATGTGTGTGTGGATCAGTGTATGTG
TTAAAATTAAGACATATGACATAACTACAGGTTTTGGGTTTGTTATTGTTTTTATCATCCTGTTCTTACTT
ACTAACAGGTGTCCTCCAGTTTACGATCCGACCTTTTGGAAACGTAC

**MdMIR156a2***

TTTTCCTTTCTCTTCGATCTGTGATTAAAAATTAAAATGGCATTAAAATAATGGACGATGACCAATAGAA
GATAAACCCAGATTTATTCCATACGCGATGCAACTCCTGTTACTCCTCTCTTTTCTCTTTTTTTCCTTCTA
TATAAAGCCCCAACAGGCCTTGCAATTTCTCATAACAAAGCAAAGCAAAGCAAAACAAGGGTAAGCT
ATATTAGGGTTCCCTCGACGGTGCGGAACCGAGGATGAGTAGAGTGATAGATTGGGGTTCCCTTCCCG
GTGAGGTCGAAKAGAGCGACCCTAGAGATTAGGGTTTGAAACCCTCCAAAAAAAAAAAAAATGTAAAC
CCTCCAAAAAAGAAAAAATGTTCCAATCTCAGAGAAAAATTTGGGGGKTTTGGGGGATAAACAGCTA
GGGTTTTTTGGAGGCCTCTTTCTCTTTCTCTCTCTGTCTCTGTCTATGTGCTGCAATATTGAAGAGAACG
TTTTAAAGGGTAAAGGGCGTTGACAGAAGAGAGTGAGCACACATGGTACTTTCTTGCATGCTGGGTTC
ATGYTCGAAGCTTTGCGTGCTCACTCTCTATCTGTCACCCCCACCTATCTCTCTCTCACTCTCTGAATCA
TGTTTGTTTTGTATATTTGCGTTTACAATTTTTCATGAATTTTCTGCTTATAAATTTGCTCCCAGAAAATA
ACTACTRATCAGACGGCGTCGATGGGGGATCTAGACTGGATCATTAATTGCATCATGTTGTCGTGGTCA
CAGAGATTTATCGATTTYGACATGTCGGTCTYAAACTATGTAGGGATTGGGGATTAGGGTTTCTTAGGG
TTTTTATTTTTATTTTTTATTTATTGCTATCGAAATCCCTTTTTATTAAGCATTGAATTGGTTGCGGACTTC
AAGGCCGTATTCAGAAGCAAAGAAAAATTATCCCCGTTTGGGCAGGTGAATTTTGGTCTCTCTTGCCAT

AATTGGTACAATACCATAAGGTTGAAAGCT GAAAATTTATCTTTATGGACCA

*Sun C., Zhao Q., Liu D. D., You C. X. & Hao Y. J. Ectopic expression of the apple Md-miRNA 156h gene regulates flower and fruit development in Arabidopsis. Plant Cell Tiss Organ Cult. 112, 343-351 (2013).*

**MdMIR156a3**

AATCATGAACTGGAGGACACCTTTGAGTGAGTAAGATCAGGTAATAAAAACAATGACAAACCCAAAA
CCTGTATGTCTTATGTCTTAATTTTAATACATACGCTGATCAACACAGACATCTATAAATATATGAATATAC
ATACAACATCTCCATTTCCCAAACAAGAAAAATAAGAGCTGGGAGTAAGAGGGTATCTAGCTATCTGTT
TCATATATATATGTGTGTGTGTGTGTATATAAATATACGGTGGAGGGAAAGACACCTGGAAGAAGGAGA
ATTGGGAGAGCAGAAGGAGGCGGGGCCTGGGACTGGCCTGGTTTGATTTCCCGTTCCTTTGCAGGAA
AGAGAAGGGGCTTTGATGAGAAATTAAGGTTTTGAACGTTGACTACCAAAATTAAGTGGTGAAATAAA
GCCAGCGAGCGCGCAGATAGCAGCTGACAGAGAGAGAGGTGAGCACGCACCGCCCAAGGGTATGAT
TTTATACAATTGCTGCCTGTGTGTGCTCACTCTCTTCTGTCAACTACGCTCAAATTAACCTACAGCTCTC
TTCTTTCTTTCTAATACATACATATATATATATATAGCTAATTAGTTACACCAAGTGCAATGGTCAGCCTAG
CARCTTTCCTCCACTTAAGGAATATCATGTTTTCATTAGGGTTATGAATTTGGAATGCAGTGCGAATGCC
CTATCTACCAGGATTATTAACTATATACACTCGCGGAGTCCTTCAATTCATCAGAGGAAGGAGGGCGGT
TGATTTCTGATCTCCATTATTAATGGTGAAAGCCAGGTTTGTGGATATAAGGACTCATCATCCATCCATAT
CTCAATCGTGTCCTTGCATCCGACGTCTCAGTACCAATAGTAATTCACTAATACCACTAGTAATTCCATG
TCGAATTTATAGTAGTACTATTTGGAAAGAAAAACGCAAGGAAACTCTCTCAAAAGTGGAGTATYCAT
AGGCTCCATCATCTTATGTTTTTRACATAATTTTCATGCTAATATTTTAAAA

**MdMIR156a4**

TATTTTACTATAGTAAATCTTGAAAGAGGAAAACAAAGAAGCTAGGGCTAGAAGATTAGATCTGTGATA
CTGATAAATGGTGATGAAACCTGAGACTCAGTTATTTATCAGTGGAACTTGAGAGAATGGATGCACACT
AGTSTATACATACTGGATATCATCTTCCAAACGAGAAGAAGGGGCAWGGTCGGAGGGTCTGCCTGGGA
AAATGAAAATGCAATAATAATTGATTGAAAAAATAGATATAARGGGRATAAATAAATAAATTCAATTCTT
ATCTTCTTGAAAGAATGAAAGAAAGAGTACACCTGGAAAGTGGAAGCTGASACGGTAGTAGGTAGMA
GAACTGAGAGAGATCGAGAGGAGGAACACAGAAAGACATAATGAAAGGGAGGGCAGCGCAGGCGG
TGGGCGGTGGGCGGYGGGCGGCGTGCGGCTGACAGAAAGAGAAGTGAGCACACATCACGCAAAGGT
ATGGCTTTATACAATTGCCCGAGTGTGCTCACTCTCTTCTGTCACCACCACTCGCTACTAGTCCAGTGAT
CTACTWCTCTCCCTCATCCAYTCACCTTCTCTCTCTCTSCCTCTGTCTCTCTCTCTATCTCTCTGTCTCTC
TCTGCTACTTGGTCTTTATATTAGTATATTACACTACTTACAGCAGCTTTTTTTTCAATCTTCTATTTATTG
CCGACAACTCCATTATCTATATATCTTCTTCCTCGACTAAAATAGATATGAAGATCTACTAACACACCATA
CTCCCCACTCTACTCTAGCTAAAGTTCATTTTGTATAGAAGGGACTCGTGACTCAACTCGTCAAGAGCA
TGTACCATCGAATATGAGGTCATGAATTCAAGTGTTTTCTCTCTTGCTATCATTTTTATTAGAATTAAAAA
GTAAAAATGTTTACCTTATGTAACTTAGGCGCGTCTCTTTATATTGAACCAAAATAAAAAATTGGGTATC
AGTAAGGATTACTATGTGTTTACTAATTCTCTGAGAATCAAAACACAAAC

**MdMIR156a5***

CCTGTAGTTATGTCATATGTCTTAATTTTAACACATACACTGATCCACACACACATCTATAAATATATACA
CAACTTCTCCATTTCCCAAACAAGAAAAGTGAGAGCTGGGAATCAGAGGGTATCTAGCTATCTGTTTC
ATATATGTGAGATAGATGCATATAGGGTTGGGAGAAAAACACCTGGAAAGAGAATTGGGAAAGTAGAA
GGGGCTGAGCCTGGGACTGGCCTGGTTTAATTTCCCATTCCTTTGCGGGAAGAGAAGGGGCGTTGATG
AGAAATTAAGGTTTTGAACGTTGACGATCAAAATTAAGTGGTGAAATAAAGCCAGCGAGCGCGCAGAT

AGGAGCTGACGGAGAGAGAAGTGAGCACGCACCGCCCAAAGGTATGATTTTATACAATTGCCGCCTG
TGTGTGCTCACTCTCTTCTGTCAACTACGCTCAGCTTAACCTACAGGTTTCTTTCTGCTTTCCAATATAC
ACAAATATACATATATACATATATATATAGCTAATTAGTTACACCACCMAGTGCCATGGTCAGCCTAGCAG
CTTTCCTTTCASTTGAGTCATATCACGTTTTTATTAGGGTTTTGAATTCGGAATGCTCYGTGAATGCCCTT
ATCTACCAGGATAATTCACTATGTATACGCGCGGAATCTTTCAATTCATCAGGACTTGAGCAAGGGGGC
CTGATTTCTGGTCTCCATTATTGATGGTGAAAGCCTGGTTGGTGGATAAAAGGACTGCCATCGATCCAT
CCACATCTGAATCATGTCCCGACATTTAAGTACCAAAAGTAATTTACTAGCACCACTAGCAATTASGTGT
CCAATTTGTAGCAGTRCCAGTGCTTTGGAAAGAAAAAGAGTAATTACATTTTTCACCTTCATATTTRAG
TGCAATTACTACCTCTTACAATAACTTTTAAAAC

**MdMIR156a6***

GAGAGACAGACAGTAACACAGAGACGGAGAGAGAGAGAGGGAGAGAGAGAGGGTCGTAGATAGCA
GCAGTACAGCTTATTTTGGTATTGGGGTTTGTGGAGGTTGTTCCCATTTTTTCTTCGTTTTGTCTTTATTT
TTCGCCATTGAGCGAAACCCTAAATTTATTTTACACCAAAATAGTATTACTCGTAGATTTTTCTGACATT
TTGGTATCTTTCCTTATATGGGTTTTTGTTTTTGTTTCTTCCTGGGTTCTCAGATCCGACTCTTTTCTATCA
GTCTTTTTATTACTTTCTTGCTTTGCCGAACCAAGGAATCCCTTAGATATAATGCAAAATGGATCTTGCT
CTATCRTTGATCAGAGATTCTCTATGAACAATACGAATCGGAGTTCTGCTTTCTTCACAAACTTTTTCTT
CTTATTAATCAAAAGAAATTGGCGATTGGGTAGTTATATTATTGTGGTGAGTGTTAATTTGGTTGGGACT
TAGGGTAGAAATTGACAGAAGAGAGTGAGCACACAGAGGCACTTGCCATAAAGCGTATGCCATTGCTT
TTGCGTGCTCATGTCTCTTTCTGTCAGCTTCCCCATTTCCACTGCCGGATTAATATTAAGTTGTTCATTTT
TAATTTCCTTCTCATCCCCATCCTTCTTCTGGTCTTTCTCTTTAATTTGTTTCACCAGTTCCCCTCTCTTCT
CTCTCTCTCTCTCAAACTCTTTAATTTATGGTGCTTAATTAACTGCTCAAAAGGGAATTCAGACTTCAAA
GTAAACTGAATCCCTAAGCCTAGCGTAGCTAAGAGAGCATGCTCCTGTTACTCTTTTGAAATCGGTAAG
TGATTATTTGTATTATATCAATTATTTTATGTTATCTTATCAGAAACTGGTGAAATGAGAGCGTGACTTTT
GATAGTGTTTTATATATAAGAATGTGGATATATCTGTTTACTGTGCGGAATTAGCGGCAAAAACCAGGTT
ATCAAGCATAACATTATTACGGGACCATCATTCACGCTCTC

**MdMIR156a7**

TGGTATTGTACCAATTATGACAAGAGAGACCAAAATTCACCTGCCTACCAAATGGGGATAATTTTTCAT
TGCTTCTGAATACGGACTTGAAGTCAGCAACAAATTTAATGCCTAATATAAAGGGATTTCGATAGCAAT
AAATAAAAAATAAAAATAAAAACCCTAAGAAACCCTAATCCCTAAACCCTACATAGTTCGAGACCGAC
ATGTCGAAATYGATAAATCTTTGTTACCACGACAACATGATGGAATTAATGATCCAGTCTAGATCCCCCA
TAGACCCCGTCTGATCAGTAACTATTTTCTGGGAGTAAATATTATCAAGCTTGATCAAGCTTGGTTAACA
AATTTATAAGCAGAAAATTCAAGAAAAATCGTAAACGCAAATATACAAAACAAACATGATTCAGAGAG
TGAGAGAGAGATAGGTGGGGGCGACAGATAGAGATTAAGCACGCAAAGCTTCGAGCATGAACCCAGC
ATGCAARAAAATACCATGTGTGCTCACTCTCTTCTGTCACCGCCTTTTACCCTTTAAAACCTTCTCTTCA
ATATTGCAGCACAGAGAGAGAGAGAGAGAGAAAGAGAGACCTCCAAAAAACCCTAGCTGTTTATCCCCA
GCCCCCCAAATTTTCTATGAGATTGGAACATTTGTTTTTTTTTTTTTTTTTTTTTTGGAGGGTTTCAAACC
CTARTCTCTAGGTCGCTCTATTCGACCTCACCGGAAGGAACCCCAWTCTATCACTCTACTCACCCTCGA
TCYYWCACCGTTGAGGA

**MdMIR156a8**

TTTTAAAATATTAGCATGAAAATTATGTTAAAAACATAAGATGATGGAGCCTATGGATACTCCACTTTTG
AGAGAGTTTCCTTGCGTTTTTCTTTCCAAATAGTACTACTATAAATTCGACATGGAATTACTAGTGGTAT

TAGTGAATTACTATTGGTACTGAGACGTCGGATGCAAGGACACGATTGAGATATGGATGGATGATGAGT
CCTTATATCCACAAACCTGGCTTTCACCATTAATAATGGAGATCAGAAATCAACCGCCCTCCTTCCTCTG
ATGAATTGAAGGACTCCGCGAGTGTATATAGTTAATAATCCTGGTAGATAGGGCATTCGCACTGCATTCC
AAATTCATAACCCTAATGAAAACATGATATTCCTTAAGTGGAGGAAAGYTGCTAGGCTGACCATTGCA
CTTGGTGTAACTAATTAGCTATATATATATATATATGTATGTATTAGAAAGAAAGAAGAGAGCTGTAGGTTAA
TTTGAGCGTAGTTGACAGAAGAGAGTGAGCACACACAGGCAGCAATTGTATAAAATCATACCCTTGGG
CGGTGCGTGCTCACCTCTCTCTCTGTCAGCTGCTATCTGCGCGCTCGCTGGCTTTATTTCACCACTTAAT
TTTGGTAGTCAACGTTCAAAACCTTAATTTCTCATCAAAGCCCCTTCTCTTTCCTGCAAAGGAACGGGA
AATCAAACCAGGCCAGTCCCAGGCCCCGCCTCCTTCTGCTCTCCCAATTCTCCTTCTTCCAGGTGTCTT
TCCCTCCACCGTATATWTATAYACACACACACACACATATATATATGAAACAGATAGCTAGATACCCTCTT
ACTCCCAGCTCTTATTTTTCTTGTTTGGGAAATGGAGATGTTGTATGTATATTCATATATTTATAGATGTCT
GTGTTGATCAGCGTATGTATTAAAATTAAGACATAAGACATACAGGTTTTGGGTTTGTCATTGTTTTTATT
ACCTGATCTTACTCACTCAAAGGTGTCCTCCAGTTCATGATT

**MdMIR156a9**

TGGTATTGTACCAATTATGACAAGAGAGACCAAAATTCACCTGCCTACCAAATGGGGATAATTTTTCAT
TGCTTCTGAATACGGACTTGAAGTCAGCAACAAATTTAATGCCTAATATAAAGGGATTTCGATAGCAAT
AAATAAAAAATAAAAATAAAAACCCTAAGAAACCCTAATCCCTAAACCCTACATAGTTCGAGACCGAC
ATGTCGAAATYGATAAATCTTTGTTACCACGACAACATGATGGAATTAATGATCCAGTCTAGATCCCCCA
TAGACCCCGTCTGATCAGTAACTATTTTCTGGGAGTAAATATTATCAAGCTTGATCAAGCTTGGTTAACA
AATTTATAAGCAGAAAATTCAAGAAAAATCGTAAACGCAAATATACAAAACAAACATGATTCAGAGAG
TGAGAGAGAGATAGGTGGGGGCGACAGATAGAGATTAAGCACGCAAAGCTTCGAGCATGAACCCAGC
ATGCAARAAAATACCATGTGTGCTCACTCTCTTCTGTCACCGCCTTTTACCCTTTAAAACCTTCTCTTCA
ATATTGCAGCACAGAGAGAGAGAGAGAGAGAGAAAGAGAGAGGCCTCCAAAAAACCCTAGCTGTTTATC
CCCAGCCCCCCAAAAATTTTCTATGAGATTGGAACATTTTGTTTTTTTTTTTTTTTTTTTTTTGGAGGGTT
TCAAACCCTARTCTCTAGGGTCGCTCTATTCGACCTCACCGGGAAGGGAACCCCAATCTATCACTCTAC
TCACCCTCGGATCCTCACCGTTGAGGRAACCCTAATAGCTTACCCTTGTTTTGCTTTGCTTTGCTTTGAT
ATGAGAAATTGCAAGGCCTGTTGAGGCTTTATATAGAAGGAAAAAAAGAGAAAAGAGAGGAGTAGCA
GGAGTTGCATCGCGTATGGAATAAACTGGGTTTGYCKTCTATTGGTCATCGTCCATTATTTTAATGCCAT
TTTAATTTTTAATCACAGATCGAAGAGAAAGGAAAAATAAAGTCGTCTAGAACTT

**MdMIR156a10**

ATTCTTTACAATTTTAAATTCAATAAAAATTAATAAAACAGGAAAAATTAAATACATTAAAATAACTTAT
CTTGTATTTTCATTCCTTTGTGATTGTGAAGTGCAGTGAGCCACGTATGGTATGGTACGTCGGTCAAATC
ATAAACTGAAGGACACCTGTYACTGAGTAAGATCAGGATAATAAAAACAATGACAAACCCAAAACCTC
TATCATCAAGTCAAATGTCTTGATTTTAAACACCAGGAAGGAGAATTGGGAGAAAACTCCTGGAAGGA
GAATTGGGAGAGCAGAAGGGGCGGGGCCTGGGACTGGCCTGGTTTAATTTCCCATTCCTTTGCGGGA
AGAGAAGGGGCGTTGATAAGAAATTAAGGCTTTGAACGTTGACGATCAAAATTAAGTGGTGAAATAA
AAGCCAGCGAGCGCGCAGATAGCAGCTGACAGAGAGAGAGGTGAGCACGCACCGCCCAAAGGTATG
ATTTTATACAATTGCTGCCTGTGTGTGCTCACTCTCTTCTGTCAACKACGCTCAGCTTAACCTACAGTTC
TCTTCCTTCTTTCTAATATATACAAATATACATATATATACAGCTAATTAGATACACCAAGTGCAATGGTCA
GCCTAGCAGCTTTCCTTCACTTAAGGAATATCATGTTTTTATTAGGGTTTTGAATTCGGAATGCTGCCCG
AATGCCCTATCTACAGGATAATTCACTATATACACAAGCGGAGTCTTTCAATTCATCAGACTTGAGGAA
GGAGGGGGGCTGATTTCAGATCCCCATTATTAATGGTGAAACCCGTGAAAGCCAGGTTGGTGTATATAA

GGACTCTCATCATCCATCCACATCTGAATCGTGTCCGTGCATTCGACGTCTAATTACCAATAGTAATTCA
CTAATACCACTAGTAATTACGTGTTCAATTGTAGTAGTACTAGTGCTTTGGGAAGAAAAATGTYGGGAG
ACTCAAAAGTTGGCTGTCCATAAGTTCCTTCATCCCATGTTTTTGTCACATTAAC

**MdMIR156a11**

CGGACACCTGTACGTCCCGTTCTGCATGATAGAGAGAGAAAAAGAAAGGCAGGKAGGAGATAAGATC
GTAAATAAGAGCAGTCAATGAAGAGAGAGAGACAGACAGTAACACAGAGACACAGAGAGAGAGAG
AGGGTAGTACATAGCAGCAGTACAGCTTTCTTTTGGTATTGGGGTTTGTGGAGGTTGTTCCTTATTTTTT
CTTCGTTTTGTCTTTCTTTTTTGCCATTGAGCGAAACCCTAAATTTATTTTACACAAAAATAGTATTACTC
GTAGATTTTKCTGACATTTTGGTACCTTTCCTTATATGGGTTTTTATTTTTCTTTCTTTCTGGGTTCTCAGA
TCTGACTCGTTTCAATCAGTCTTTTTATTACTTTCTTGCTTTCAAGTTCTGCTTTCTTCACAAACTTTTTC
TTCTTATTAATCAAAAGAAATTGGCAATTGGGTAGTTATATTATTGTGGTGWGTGTTAATTTGGTTGGGA
CTTAGGGTAGAAATTGACAGAAGAGAGTGAGCACAYAGAGGCACTTTGCCATAAAGCGTATGCCATTG
CTTTTGCGTGCTCATGTCTCTTTCTGTCAGCTTCCCCATTTCCACYGCCGGATTAATATTAAGTTSTTCAT
TTTTTCCTTCTCATCCCCATCCTTCTTCTGGTCTTTCTCTTTAWTTTRTTTCACCAGTTCCCCTCTCTTCT
CTCTCTCTCAAAATCTTTTATTTATGGTGCTTAATTAACTGCTCAAAAGGGGATTCCGACTTGAAWGTA
AACTGAATCCCTAAGCCTAGCGTAGCTAAGAGAGCATGCTCCTGTTACTCTCTTGAAATCGGTAAGTGA
TTATATGTATATTATATCAATTATTTTATGTTATCTTATCAGAAACTGGTGAAATGAGAGCGTGACTTTTGA
TATTGTTTTATATATAAGAAKGTGGATGTATCTGTTTACTGTGCGAAATTAGCGGCAAAAAACCAGGTTA
TCAACGCATAGCATTAGTACGGGACCATCATTCACGCTCTCTGG

**MdMIR156a12***

TTACTATAGTAAATCTTGAAAGAGGAAAACAAAGAAGCTAGGGCTAGAAGATTAGATCTGTGATACTG
ATAAATGGTGATGAAACCTGAGACTCAGTTATTTATCAGTGGAACTTGAGAGAATGGATGCACACTAGT
STATACATACTGGATATCATCTTCCAAACGAGAAGAAGGGGCAWGGTCGGAGGGTCTGCCTGGGAAAA
TGAAAATGCAATAATAATTGATTGAAAAAATAGATATAARGGGRATAAATAAATAAATTCAATTCTTATC
TTCTTGAAAGAATGAAAGAAAGAGTACACCTGGAAAGTGGAAGCTGASACGGTAGTAGGTAGMAGA
ACTGAGAGAGAGATCGAGAGGAGGAACACAGAAAGACATAATGAAAGGGAGGGCAGCGCAGGCGG
TGGGCGGTGGGCGGYGGGCGGYGGGCGTGCGGCTGACAGAAAGAGAAGTGAGCACACATCACGCA
AAGGTATGGCTTTATACAATTGCCCGAGTGTGCTCACTCTCTTCTGTCACCACCACTCGCTACTAGTCC
AGTGATCTACTACTCTCCCTCATCCATTCACCTTCTCTCTCTCTGCCTCTGTCTCTCTCTCTATCTCTCTG
TCTCTCTCTGCTACTTGGTCTTTATATTAGTATATTACACTACTTACAGCAGCTTTTTTTTCAATCTTCTAT
TTATTGCCGACAACTCCATTATCTATATATCTTCTTCCTCGACTAAAATAGATATGAAGATCTACTAACAC
ACCATACTCCCCACTCTACTCTAGCTAAAGTTCATTTTGTATAGAAGGGACTCGTGACTCAACTCGTCA
AGAGCATGTACCATCGAATATGAGGTCATGAATTCAAGTGTTTTCTCTCTTGCTATCATTTTTATTAGAAT
TAAAAAGTAAAAATGTTTACCTTATGTAACTTAGGCGCGTCTCTTTATATTGAACCAAAATAAAAAATTG
GGTATCAGTAAGGATTACTATGTGTTTACTAATTCTCTGAGAATCAA AACACAAAC

**MdMIR156a13**

TTTGTGTCCACAGAGAAATTTTCAACTTTCAGCCTACGGTATAAATTGCACCAATTAGGGCGAGAGAG
CAATATTCACCTGGCTACCAAGTATAGATAAATACTTTTTGGAAAACGGCCTTGAAGTCAGCAAATTAA
TGATAAATTTAATGCCTAAAATTAAAGGATTTTCGACAGCAAAAAACTAAAAATAAAAATAAAAACCCT
AGAAACCCTAATCACTAATCCGTACATAGTTTGAGACCAACATGTCAAAATCAAATAAATCTCTGTGAC
CACGACAACAATATGCAATGATCCAGTCTAGATTCCCCATCGATTAGGACTGATCAGTGGTAATTTCCT

GGGTGTAAATTTATATGCAGAAATTTCATGAAAAATTGTAAACAGGCAAAAATACAAAACAAACATGA
TTCAGAGAGAGAGAGAGGTGGGTGGACAGATAGAGAGTAAGCACACAAAGCTTCAAGCATGAACCC
AGCATGCAAGAAAATACCATGTGTGCTCACTCTCTTCTGTCACCGTCCTTTACCCTTTAATTATTTGAAC
CTCCTTCATCGATATTGCACCAGATAGAGAGAGGGGCATCCCAAAACCTTAGCTGTTAACCCCCAAATG
CTTTATGAGATTGGAACATTTATTTGCTTTTTTAGGGAGAGGATTTTGAACCCTAGATCTAGCTACTCCT
ATCTATCGCTCTGTTCGACCTCACCGGGAAGGGAACACCTATCTATCACTCTACTCACCCTAGCTTGGT
CCCTCACCGGTGAGGGAACCCTAATAGCTTAACTTTGATTTGCTTTGATATGAGAAACTGCAAGGCCTG
TTGGGGCTTTATATAGAAGGAAAAGGAGAGAAAAGAGAGGATTGCATCACGTATGGAATAAATGTGGG
TTTATCTTCTATTGGTCATCTTCGATTATTTAATGCCATTTTAATTCTTAATCACAGATCGAAGAGAATGG
AAAAAAAAAGTCGTCTAGAACTTTATCTCTTCCCATCCGATATGTTCAAACCCCAA

**MdMIR156a14**

CATACTATAGAGATAGAGCGTGAATAATGGTCCCATGATATGCTATATGCTTGATAACCTGGTTTTTGCCG
CTAATTTCTCACAGTAAACAGGTATATGTACCTTCTCATATATAAAACAATATCAAAAGTCACGCTCTCA
AGGTAATAGTTTCAGCTGCTTAGATAACATAAAAACAATTGATACAATATGCATATAATCACTTACCGAT
TTCAAAGGAGTAACAGGAGCATGCTCTCTTAGCTAGGCTAGGCTTAGGGGTTCAGTTTCTTTGAAGTT
TGAATCCCCTTGAGCAGTTAATTAAGCACCGTAAATAAAACATTTTGAGAGAGAAGAGAACTGGTGAA
ACAAATTAAAGAGGGAGACGAGAAGAAGGGAGGGGGTGTGAAGGAAGAAATGAAAAAATTAATATT
AATCCGGCAGTGGAAATGGGGAAGCTGACAGAAAGAGACATGAGCACGCAAAAGCAATGGCATACG
CTTTATGGCAAGTGCCTCTGTGTGCTCACTCTCTTCTGTCAATTTCTACCCTAAGTCCAACCAGATTAAA
TTTCTAAACACAAGAATATATAACTACCCAATACTGAATTTTTGATAATTAATAAGAAAAAAAAAGATTG
GGAAGAAAGCAGAACTTGAAAGAGAGAGATAAATAAAAAGATCGGTAGAAATGAGTCAGATCTGAG
AACCCAGAAGGAGAGAATAAAATCCCATATAAGGAAAGATACCAAAATGTCGGAATAATCAACAAATA
GTAATCTTTTGGTGTGAAGGAAATTTAGGGTTTTGTTCAATGATAAAAAGTAAATAAAAGAGAGTTTGC
CAAAAAAAAAAGTAAATAAAAGAGAAGAAAATATGAAAACAACCACCACAAACCCCAATAAGAAAA
GAAGCTGTACTGCTACTACTACTGCTGGCTATCTGCTACCCTTTCTTTCTCTCTCTGTGTGTCTCTGTGT
TTCTGTATGTCTCTTTCTTCATTGACTCTCTTATTTATGATCTTAT CTCATCCTTGCCCTTC

**MdMIR156a15**

ACATATGGTATGGGATCATGGGAAAAGAWWAAAAAAGTGATAAAGTTCTAGATGACTTTTTTCCATT
CTCTTCGATCTGTGATTAAGAATTAAATGGCATTAAAATATTTAAAATAATGAACGATGACCAATAGAAG
ATATACCCACATTTATTCCATACGTGATGCAACTCCTCTCTTCTCTCTTTTTCCTTCTATATAAAGCCCCA
ACAGGCCTTGCAATTTCTCATATCAAAGCAAAGCTATTAGGGTTTCCTCAACGATGAGGGATCGAGGTT
GCAGTAGAGTGATAGATAGGGTTCCCTTCCCGGCGAGGTCGAATAGAGGGATGGATAGGAGTAGCTAG
ATGTAGGCTTGGAAACCCTCTCCCAAAAAAGCAAATAAATGTTCCAATCTCATATATAGAGCATTTGGG
GGTTAAACAGCTAGGGTTTTGGGAGGCCTCTTTCTCTCTGGTGCAATATCGAAGGAGGTTCAAATAATT
AAAGGGTAAAGGACGGTGACAGAAGAGAGTGAGCACACATGGTATTTTCTTGCATGCTGGGTTCATG
CTTGAAGCTTTGCGTGCTTACTCTCTATCTGTCACCATCACCTCCCTCTCTGAATCATGTTTGTTTTATAT
ATTTGTGTTTACAATTTTTCATGAATTTTTTGCATATAAATTTACACCCAGGAAATTGTTGAGAATAAGTC
TCACATTAATGGGAGGAGAGACCTTGAATAGGCTTATAAGAGGTTGAGCTACTCTCCATATGGTGGAAC
CCCAACTTTCTTCATGGCATTAGAGCAAGTTGTCACACGTGTGAAGCCAAACGGTCACACGTGCTCCA
AGTCACCCGTTGTGTTGTTCACGTGTTAGGCTTGAAAATTTGCCACACATGAGAGGGTGTGTTGGGAA
GGAGTCTTACATTGATGAGAGGAGGGACTTAGGTTCTAAGAGATTTGGCTACTTTTTATATTATCAATTG
ATTTTATGATGGAACTCAACTTTCTTAATTAGTTTTATGGTGGAACTCCTTT

**MdMIR156b1**

GGTCATTAAGTTAACCTTCCAACCCGCCCAACCCGTCCGAGTTGCATCCCTAGATAGTAATTCAGCTGT
TATATATTTGAGGAAAGTAAAAAAAGGGGGAAATTCTCAATCATGTTGTTGTTGTGCAATGACTGAAAG
GACTGAAATAAGCAAACAAACCATGCGTACGGAAGCAAAAAACATCACACACACACACACATGTGTA
AAACCTATATATGTATATATGTGTGTGTGTGTGTGTGTGTGTGTGTGTGTGTGTATGTATATTTAATTATTT
CATGCTTTCCAAGATTTATCAATCGAGATGCACTCTATATGTTAGTACTACTTGTTCATCTCGGGAAGTT
CAGACAGAATGGATAGACGATTAATATGGATTATAGAAAGGCATCATATGGCTATATAAAGGCAATAGGG
AAGGTAAGGGGCTGAAGGTGTTGACAGAAGAATAGAGAGCACAAAGGATGGAGATGCTGCCTTGTTT
TTGCATTTCCTCATCTGTGCTCTCTATCTTCTGTCAGCAAATTCACCTATGCCTCCTCCATCTACAAATAT
ATATATATATATATATATATATATATATATATATATATAGCAACAAAACTTAAAAATATTACAACTAATTGCAAGG
CAAATGAAAGATAATGGAAGAACTTAAAAGTTGAGACCCACAMACAATTTGGGTTGGGAGGGAGAG
AGGGAGAGAGAGCTGACCTTGGGATGAACTTAAACGACTTCCAATGCCACTATTTATAGGGAAGGAGT
AAGGTCTAAGCATGCATGCATGTTGGCATGTCTTATCATGCATGAAGCCGTTTTCTGTGAGATGCATAA
ACGAATAAAATGTTCCCCAAATCACGATAATTGTGTGCGTTCCTGATGCCTCACGCAAATCGGGTGTAG
TAGTCAAATGATATGGATTCATTCCAAAAGTATTAATTATAATTAATCGTGGATATACACCCAAAATTTGG
GAATTAAAAGTGCTTGTGGCAATGACTAAAATTTTCAA AATAATTCTTC

**MdMIR156b2**

TGATAATAAGCGGCTGTGAAATAAATCAACAGAGTGTTTGATAAACTTTTTTGTAAAAGTGCTTTAGGA
AAAAAAAAAACAGTCTGATAGTGGGTCTTTTCATTAAAGGAGCAATGTAGCTCCGTGTGCTTTGAAAA
AAAACCAGTTTTCCAAAGCTGCAAATAGCAGCTTCAGCATTTTCCTTTGATTTTAGCTTATTCTCACAG
CAGCTTCCAAAATAAGCCCYTTTTTTTTAGTTTACCAAACACCTAAAACCTTCACAGCTTTTTTTCATG
GGTGCTTTTTTTTTAAGCACCTCACTCCCAAACCACCCCTAAGTTCATCCCAAGATCAGCTCTCTCTCC
CTCTCTCCCTCCCAACCCAAATTGTCTTTGTGGCTCTCAACTTTTAAGTTCTTTCATTATCTTTCATTTGC
CTTGCAATTAGTGGTAATATTTTTAAGTTTTGTTGCTATATATATATATATATATATTTGTAGATGGAGGAGGC
ATAGGTGAATTTGCTGACAGAAGATAGAGAGCACAGATGAGGAAATGCAAGAACAAGGCAGCATCTC
CATCCTTTGTGCTCTCTATTCTTCTGTCAACACCTCCAGCCCCTTACCTTCCCTATTGCCTTTATATAGCC
ATATGATGCCTTTCTATAATCCATATTAATCGTCTATCCATTCTATCTGAACTTCCTTCAAGATGAACAAGT
AGTACTAACATATAGAGTGCATCTCGATTGATAAATCTTGGAAAGCATGAAATAATTAAATATACATACA
CACACACACACACACACACACACACACACACACATATATACATATATAGGTTTTACACATGTGTGTGTGTG
TGTGATGTTTTTTGCTTCCGTACGCATGGTTTGTTTGCTTATTTCAGTCCTTTCAGTCATTGCACAACAA
CAACATGATTGAGAATTTCCCCCTTTTTTTACTTTCCTCAAATATATAACAGCTGAATTACTATCTAGGGA
TGCAACTCGGACGGGTTGGGCGGGTTGGAAGGTTAACTTAATGA

**MdMIR156b3**

AAGGAAGAATTATTTTGAAAATTTTAGTCATTGCCACAAGCACTTTTAATTCCCAAATTTTGGGTGTATA
TCCACGATTAATTATAATTAATACTTTTGGAATGAATCCATATCATTTGACTACTACACCCGATTTGCGTG
AGGCATCAGGAACGCACACAATTATCGTGATTTGGGGAACATTTTATTCGTTTATGCATCTCACAGAAA
ACGGCTTCATGCATGATAAGACATGCCAACATGCATGCATGCTTAGACCTTACTCCTTCCCTATAAATAG
TGGCATTGGAAGTCGTTTAAGTTCATCCCAAGGTCAGCTCTCTCTCCCTCTCTCCCTCCCAACCCAAAT
TGTCTKTGTGGGTCTCAACTTTTAAGTTCTTCCATTATCTTTCATTTGCCTTGCAATTAGTTGTAATATTT
TTAAGTTTTGTTGCATATATATATTTTTATATATATATATATATATATATATATTTGTAGATGGAGGAGGCATAGGTG
AATTTGCTGACAGAAGATAGAGAGCACAGATGAGGAAATGCAAAAACAAGGCAGCATCTCCATCCTT

TGTGCTCTCTATTCTTCTGTCAACACCTTCAGCCCCTTACCTTCCCTATTGCCTTTATATAGCCATATGAT
GCCTTTCTATAATCCATATTAATCGTCTATCCATTCTGTCTGAACTTCCGAGATGAACAAGTAGTACTAAC
ATATAGAGTGCATCTCGATTGATAAATCTTGGAAAGCATGAAATAATTAAATATACRTATATAATCAAMA
CACACACACACACACAYATATATATATATAYATACATATATAGTGTGTGATGTTTTTTTGCTTCCGTACGCA
TGGTTTGTTTGCTTATTTCAGTCCTTTCAGTCATTGCACAACAACAGCATGATTGAGAATTTCCCCCTTT
TTTTACTTTCCTCAAATATATAACGGCTGAATTCCTATCTAGGGGCCCAACTCGGACGGGTTGGGCTGG
TTGGAAGGTTAACTCAATCTTGACCTAACTTTTACAC

**MdMIR156b4**

TGATAATAAGCGGCTGTGAAATAAATCAACAGAGTGTTTGATAAACTTTTTTGTAAAAGTGCTTTAGGA
AAAAAAAAAACAGTCTGATAGTGGGTCTTTTCATTAAAGGAGCAATGTAGCTCCGTGTGCTTTGAAAA
AAAACCAGTTTTCCAAAGCTGCAAATAGCAGCTTCAGCATTTTCCTTTGATTTTAGCTTATTCTCACAG
CAGCTTCCAAAATAAGCCCYTTTTTTTTAGTTTACCAAACACCTAAAACCTTCACAGCTTTTTTTCATG
GGTGCTTTTTTTTTAAGCACCTCACTCCCAAACCACCCCTAAGTTCATCCCAAGATCAGCTCTCTCTCC
CTCTCTCCCTCCCAACCCAAATTGTCTTTGTGGCTCTCAACTTTTAAGTTCTTTCATTATCTTTCATTTGC
CTTGCAATTAGTGGTAATATTTTTAAGTTTTGTTGCTATATATATATATATATATATTTGTAGATGGAGGAGGC
ATAGGTGAATTTGCTGACAGAAGATAGAGAGCACAGATGAGGAAATGCAAGAACAAGGCAGCATCTC
CATCCTTTGTGCTCTCTATTCTTCTGTCAACACCTCCAGCCCCTTACCTTCCCTATTGCCTTTATATAGCC
ATATGATGCCTTTCTATAATCCATATTAATCGTCTATCCATTCTATCTGAACTTCCTTCAAGATGAACAAGT
AGTACTAACATATAGAGTGCATCTCGATTGATAAATCTTGGAAAGCATGAAATAATTAAATATACATACA
CACACACACACACACACACACACACACACACATATATACATATATAGGTTTTACACATGTGTGTGTGTG
TGTGATGTTTTTTGCTTCCGTACGCATGGTTTGTTTGCTTATTTCAGTCCTTTCAGTCATTGCACAACAA
CAACATGATTGAGAATTTCCCCCTTTTTTTACTTTCCTCAAATATATAACAGCTGAATTACTATCTAGGGA
TGCAACTCGGACGGGTTGGGCGGGTTGGAAGGTTAACTTAATGA

**MdMIR156c1**

GGCTATATATAGAGGCAAGTTCTTATGTTTAATTATTTATCTGTTAGCAAAAATTATGCTTATAATAATGAA
TTAACGGATATTTAAAATCATATATCTATRCAGAGTATGGTGTGTGAGTAGAAGAGGAGTTTCACGGCA
CAAACTGTTTGTCCCCCGCAACTCATGGGCCAAAATCTGACTTCGAGACCGAAAGTTGYACTAWACCG
GGTTTGTGCCATTAAATACCTCTTCTACAACTGCGCCAGTATCTATACATATGMATTTATATATATATATAT
ATATATATATRTATATATATRTATATGTATGTATGTATGTATGTAAGGGGTAGTASACATGCTAATATGCATTG
TATACAATATATCAGATTTATATGAGCCTGAATGCGTTACCTCACCCTATTAATTGATGGTTTGCWCTGCA
GGGATGAATGCATGAGTTTTGTGGCATTAAGGGTGTATAGATGAGATGGGGAATGAGGTTAGAGTTC
ATATATGTTGACAGAAGAGAGAGAGCACAACCCGCCATCGGCTAAGGAGAGTCTTTACCGTTGCTGGG
AGTGTGCTTTCTCYTCYTCTGTCATCATCTCTTACATTCCCATCTGTCAGCACCGTCGTCTGATCTACTT
CGATCTAACCTCAAAGTAAGATATTCTCTCACAAAATTAAGGAACATTGTTATCTACAAATATATATATAT
ATATATATATATATATATATATATATATATATATATATATATATATATATAGCTCTATTAAACTAAACATGGTAAATA
CCAATATTAAACTCAACATAGTAAATACCAAAAATATATATGACAATATTTGAGAATTTCTTATATATTCAT
TAATCTTCAAAGTGGAGTATATATAGGAGTTACAGTTGATATTACATATGTACTTCACCAATATGGGACAA
TAAACTACTATTTACACTTTAACTAATGTATAACTCGCAATACTCCCCCTCAAGTTGGTGCAAAGATGTC
AC GCATGTCCAACTTGTCAAGCGAGTTGGA

**MdMIR156c2**

ATGTTGACAGAAGAGAGAGAGCACAACCCGCCATCGGCTAAGGAGAGTCTTTACGTTGCTGGGAGTG

TGCTTTCTCTTCTTCTGTCATCATCTCTTACATTCCCATCTGTCAGCACCGTCGTCTGATCTACTTCGATC
TAACCTCAAAGTAAGATATTCTCTCACAAAATTAAGGAACATTGTTATCTACAAATATATATATATATATAT
ATATATATAGCTCTATTAAACTAAACATGGTAAATACCAATATTAAACTCAACATGGTAAATACCAAAAAT
ATATATGACAATATTTAGGAATTTCTTATATATTCATTAATCTCCAAAGTAGAGTATATATAGGAGTTACAG
TTGATATTACATATGTACTTCACCAATGTGGGACAATAAACTACTATTTACACTTTAACTAATGTATAACT
CGCAACACTCCCTCTCAAGTTGGTGCAAAGATGTCACGCATGCCCAACTTGTCAAGCGAGTTGGAAA
ACACACTATTAGACACAGCCTTAGTAAGAGCATCAACAAGTTGATCTTCAGATCTCACA

**MdMIR156c3**

AGTTAAAGTGAGATATTAGAGACAGCCAACAATTTCGTAGAATTCAGTAGGAGAGGAAAGCAAGGAA
CCAGCTGACAGTGAAAGAAGTGTTGTGAGTTATATATTAGTTAGAGTGTAAATAGTAGTTTATTGTCCC
ACATTGGTGAAGTACATATGTAATATCAATTGTAACTTCTATATACACTCCACTTTGGAGATTAATGAATA
TATAAGAAATTCCTAAATATTGTCATATWTATTTTTGGTATTTACCATGTTWAGTTTAATAKTGSTTTAKTT
ATWTAYTWWRTTTAKTTTAATAKAKMTATATATATATATATATATATATATATATTTGTAGATAACAATGTTCC
TTAATTTTGTGAGAGAATATCTTACTTTGAGGTTAGATCGAAGTAGATCAGACGACGGTGCTGACAGAT
GGGAATGTAAGAGATGATGACAGAAGAAGAGAAAGCACACTCCCAGCAACGTAAAGACTCTCCTTAG
CCGATGGCGGGTTGTGCTCTCTCTCTTCTGTCAACATATATGAACTCTAACCTCATTCCCCATCTCATCTA
TACACCCTTAATGCCACAAAACTCATGCATTCATCCCTGCAGWGCAAACCATCAATTAATAGGGTGAG
GTAACGCATTCAGGCTCATATAAATCTGATATATTGTATACAATGCATATTAGCATGTSTACTACCCCTTAC
ATACATACATACATACATAYAYATATATATAYATATATATATATATATATAKCATATRTATASATACTGCKCAK
WTGTAKAARWRSTATYTAATGGCACAAACCCGGTWTAGTRCAACTTTCGGTCTCGAAGTCAGATTTTG
GCCCATGAGTTGCGGGGGACAAACAGTTTGTGCCGTGAAACTCCTCTTCTACTCACACACCATACTCT
GYATAGATATATGATTTTAAATATCCGTTAATTCATTATTATAAGCATAATTTTTGCTAACAGATAAATAATT
AAACATAAGAACTTGCCTCTATATATAGCCAAAAATTAAC

**MdMIR156c4**

GAAAGTAAAAAAAGTTGGAGAAACACAATTAATAAATAAATTACTTGTATAAATATTACAACACACACA
TAATATTGCAAACTAAAAGAGACAGACGCTATTAGAAGCTGTCGACCTAACATAAAAAACCRCCATAAT
CTTGCTAGAATTGTCTAGCATAGTTWACATATATGCATGCACCGGCCACTTCCATTGCTTGCTAGAGTTC
TCTAGCAAAATCTCTGCATGTTTGAACTAGAAAAATCTGTAGTTCCTGCACCTGACCAAAATGCAGTCT
ACAGAAGCTACTGACTTTGGGCYGGTGTTGATCTTTCAACAGGGAAGACATGCTWATATGCGTTTGCA
CACATTTTATGAGATTTATATGATYMTGAATGCATTATCTCATTACATCTAAAGCCTGGCTATTAACTGAT
GGCTAACTTGCACTGCAGGGATGAATGCATGAGTTGTGGCATCGAGGGTGGTGGATGTGATGGGGATG
AGGTTAGAGATCATGTTGACAGAAGAGAGAGAGCACAACCCRCCATCGGCTAAAGAGAGTCTTTTCC
GTTGTTGGGAGTGTGCTCTCTTTTCTTCTGTCATCATCTCCTACATTCCCGTCTCTCAGCACYGTCTGAT
CTCTGTCGATCTATARCCTSAAAGTAATTAAGGTATTCTCTCACCAGGGACWTTGTTACATACAAATTTA
TAGTTCAACATTATATACAGCTAATAAGGAAATCACTTAATTTGTGGATCYTGTGTGTTTGTGTGTGTAG
TATTACTTGTGTGCAAAACYGGGGAAATTTCTTACGCAGATGATTCTTGTWGCAGCGGTAATTAACCA
GCYGAATTMTTTTTTGGAGGAATAAATTTTACGAATTATTTTGCAGCTCTTGATCAAATATTTATTWTGG
TCAACTTATAAGCGACGCTGGTTTACTACTAATCTTGGTTTCTACTTGTACAACATTCAAATGTTATTCG
GAAACTTGATCGGTGTTGTAAACCGTGACAGCTTAGTGATCGACATAGTTT

**MdMIR156d1**

AGGAAGAATTATTTTGAAAATTTTAGTCATTGCCACAAGCACTTTTAATTCCCAAATTTTGGGTGTATAT

CCACGATTAATTATAATTAATACTTTTGGAATGAATCCATATCATTTGACTACTACACCCGATTTGCGTGA
GGCATCAGGAACGCACACAATTATCGTGATTTGGGGAACATTTTATTCGTTTATGCATCTCACAGAAAA
CGGCTTCATGCATGATAAGACATGCCAACATGCATGCATGCTTAGACCTTACTCCTTCCCTATAAATAGT
GGCATTGGAAGTCGTTTAAGTTCATCCCAAGGTCAGCTCTCTCTCCCTCTCTCCCTCCCAACCCAAATT
GTCTKTGTGGGTCTCAACTTTTAAGTTCTTCCATTATCTTTCATTTGCCTTGCAATTAGTTGTAATATTTT
TAAGTTTTGTTGCATATATATTTTTATATATATATATATATATATATATATTTGTAGATGGAGGAGGCATAGGTG
AATTTGCTGACAGAAGATAGAGAGCACAGATGAGGAAATGCAAAAACAAGGCAGCATCTCCATCCTT
TGTGCTCTCTATTCTTCTGTCAACACCTTCAGCCCCTTACCTTCCCTATTGCCTTTATATAGCCATATGAT
GCCTTTCTATAATCCATATTAATCGTCTATCCATTCTGTCTGAACTTCCGAGATGAACAAGTAGTACTAAC
ATATAGAGTGCATCTCGATTGATAAATCTTGGAAAGCATGAAATAATTAAATATACRTATATAATCAAMA
CACACACACACACACAYATATATATATATAYATACATATATAGTGTGTGATGTTTTTTTGCTTCCGTACGCA
TGGTTTGTTTGCTTATTTCAGTCCTTTCAGTCATTGCACAACAACAGCATGATTGAGAATTTCCCCCTTT
TTTTACTTTCCTCAAATATATAACGGCTGAATTCCTATCTAGGGGCCCAACTCGGACGGGTTGGGCTGG
TTGGAAGGTTAACTCAATCTTGACCTAACTTTTACAC

**MdMIR156d2**

GATAATAAGCGGCTGTGAAATAAATCAACAGAGTGTTTGATAAACTTTTTTGTAAAAGTGCTTTAGGAA
AAAAAAAAACAGTCTGATAGTGGGTCTTTTCATTAAAGGAGCAATGTAGCTCCGTGTGCTTTGAAAAA
AAACCAGTTTTCCAAAGCTGCAAATAGCAGCTTCAGCATTTTCCTTTGATTTTAGCTTATTCTCACAGC
AGCTTCCAAAATAAGCCCYTTTTTTTTAGTTTACCAAACACCTAAAACCTTCACAGCTTTTTTTCATGG
GTGCTTTTTTTTTAAGCACCTCACTCCCAAACCACCCCTAAGTTCATCCCAAGATCAGCTCTCTCTCCC
TCTCTCCCTCCCAACCCAAATTGTCTTTGTGGCTCTCAACTTTTAAGTTCTTTCATTATCTTTCATTTGCC
TTGCAATTAGTGGTAATATTTTTAAGTTTTGTTGCTATATATATATATATATATATTTGTAGATGGAGGAGGCAT
AGGTGAATTTGCTGACAGAAGATAGAGAGCACAGATGAGGAAATGCAAGAACAAGGCAGCATCTCCA
TCCTTTGTGCTCTCTATTCTTCTGTCAACACCTCCAGCCCCTTACCTTCCCTATTGCCTTTATATAGCCAT
ATGATGCCTTTCTATAATCCATATTAATCGTCTATCCATTCTATCTGAACTTCCTTCAAGATGAACAAGTA
GTACTAACATATAGAGTGCATCTCGATTGATAAATCTTGGAAAGCATGAAATAATTAAATATACATACAC
ACACACACACACACACACACACACACACACATATATACATATATAGGTTTTACACATGTGTGTGTGTGT
GTGATGTTTTTTGCTTCCGTACGCATGGTTTGTTTGCTTATTTCAGTCCTTTCAGTCATTGCACAACAAC
AACATGATTGAGAATTTCCCCCTTTTTTTACTTTCCTCAAATATATAACAGCTGAATTACTATCTAGGGAT
GCAACTCGGACGGGTTGGGCGGGTTGGAAGGTTAACTTAATGA

**MdMIR156d3**

GGTCATTAAGTTAACCTTCCAACCCGCCCAACCCGTCCGAGTTGCATCCCTAGATAGTAATTCAGCTGT
TATATATTTGAGGAAAGTAAAAAAAGGGGGAAATTCTCAATCATGTTGTTGTTGTGCAATGACTGAAAG
GACTGAAATAAGCAAACAAACCATGCGTACGGAAGCAAAAAACATCACACACACACACACATGTGTA
AAACCTATATATGTATATATGTGTGTGTGTGTGTGTGTGTGTGTGTGTGTGTGTATGTATATTTAATTATTT
CATGCTTTCCAAGATTTATCAATCGAGATGCACTCTATATGTTAGTACTACTTGTTCATCTCGGGAAGTT
CAGACAGAATGGATAGACGATTAATATGGATTATAGAAAGGCATCATATGGCTATATAAAGGCAATAGGG
AAGGTAAGGGGCTGAAGGTGTTGACAGAAGAATAGAGAGCACAAAGGATGGAGATGCTGCCTTGTTT
TTGCATTTCCTCATCTGTGCTCTCTATCTTCTGTCAGCAAATTCACCTATGCCTCCTCCATCTACAAATAT
ATATATATATATATATATATATATATATATATATATATAGCAACAAAACTTAAAAATATTACAACTAATTGCAAGG
CAAATGAAAGATAATGGAAGAACTTAAAAGTTGAGACCCACAMACAATTTGGGTTGGGAGGGAGAG
AGGGAGAGAGAGCTGACCTTGGGATGAACTTAAACGACTTCCAATGCCACTATTTATAGGGAAGGAGT

AAGGTCTAAGCATGCATGCATGTTGGCATGTCTTATCATGCATGAAGCCGTTTTCTGTGAGATGCATAA
ACGAATAAAATGTTCCCCAAATCACGATAATTGTGTGCGTTCCTGATGCCTCACGCAAATCGGGTGTAG
TAGTCAAATGATATGGATTCATTCCAAAAGTATTAATTATAATTAATCGTGGATATACACCCAAAATTTGG
GAATTAAAAGTGCTTGTGGCAATGACTAAAATTTTCAA AATAATTCTT

**MdMIR156d4**

GATAATAAGCGGCTGTGAAATAAATCAACAGAGTGTTTGATAAACTTTTTTGTAAAAGTGCTTTAGGAA
AAAAAAAAACAGTCTGATAGTGGGTCTTTTCATTAAAGGAGCAATGTAGCTCCGTGTGCTTTGAAAAA
AAACCAGTTTTCCAAAGCTGCAAATAGCAGCTTCAGCATTTTCCTTTGATTTTAGCTTATTCTCACAGC
AGCTTCCAAAATAAGCCCYTTTTTTTTAGTTTACCAAACACCTAAAACCTTCACAGCTTTTTTTCATGG
GTGCTTTTTTTTTAAGCACCTCACTCCCAAACCACCCCTAAGTTCATCCCAAGATCAGCTCTCTCTCCC
TCTCTCCCTCCCAACCCAAATTGTCTTTGTGGCTCTCAACTTTTAAGTTCTTTCATTATCTTTCATTTGCC
TTGCAATTAGTGGTAATATTTTTAAGTTTTGTTGCTATATATATATATATATATTTGTAGATGGAGGAGGCAT
AGGTGAATTTGCTGACAGAAGATAGAGAGCACAGATGAGGAAATGCAAGAACAAGGCAGCATCTCCA
TCCTTTGTGCTCTCTATTCTTCTGTCAACACCTCCAGCCCCTTACCTTCCCTATTGCCTTTATATAGCCAT
ATGATGCCTTTCTATAATCCATATTAATCGTCTATCCATTCTATCTGAACTTCCTTCAAGATGAACAAGTA
GTACTAACATATAGAGTGCATCTCGATTGATAAATCTTGGAAAGCATGAAATAATTAAATATACATACAC
ACACACACACACACACACACACACACACACATATATACATATATAGGTTTTACACATGTGTGTGTGTGT
GTGATGTTTTTTGCTTCCGTACGCATGGTTTGTTTGCTTATTTCAGTCCTTTCAGTCATTGCACAACAAC
AACATGATTGAGAATTTCCCCCTTTTTTTACTTTCCTCAAATATATAACAGCTGAATTACTATCTAGGGAT
GCAACTCGGACGGGTTGGGCGGGTTGGAAGGTTAACTTAATGA

**MdMIR156e1**

AGTACCCTTGTGACCGAGTTGCTAAAACTAGGATTATGTGTATCTGCCTTTTTTAATAGGAACTTTAACG
AAAAACTTCAGGTACTGTTTACTTTAATAAAAAACCATATTTTTACGTTAAAAAGTCAATCATGGTACTA
TTCACTTTACCTTTTATTTTGTCATTATCATTAAAACTCAAAATTTTCAAACTCTTTTCATTAGTTTTTTTT
TTAATTCTTTTCCAAGAAGAATATTTTTGCTTGCCATCTTATTTATTTTATAAATTTAAATTTTAAGATATAT
GTAATCAAGACATAAATTTAAAATTTAAAATTTATTTATAGAATAGTGAATATTATCACTAGAGTGTTCTC
CTATAAATAGGGACCATTGGTGGAGGTCTGTTGAAGTATCTTCTATGTCTGCAACTCTATTATTTAAACA
TTATTATAATATTAAATTTAGGGTAAGGAGTGGGGAACTGGGAGAGAGAAAGAGCAGGGAGTGATGAT
ACATTGTTGACAGAAGATAGAGAGCACAGATGGATGACTTGCAATTGACTATGCATCTCACTCCTTTGT
GCTCTCTATGCTTCTGTCATCACCTCGTTATATCTAGCTTCCTTGCCTCTCTCCACACCCGTGCACCTCC
CATATTAATCTAATTCACTAGTTAATCAGCTGATATATGCTTCCCTTTCAATTGCTTATATATTGTTTAGATT
TTGTAATTTCAGTATTAATCTGAACTGATTTTGGGTATATATTCATCAATTTATTGATTTCTCAATTTTGGA
TCTGACTTGTATATATAGTTGGATTTTCCTTTTTCCTCTTTTGTAGGATCTTATGTTTTACAGGTTTGATTG
AAGTAGTAGTACTGGGCAAGCAGATCTATCAGAGCCGCAGGAACACAGGTTCTTATAAACATATTTATA
AGTATATACACATATATATGTGTGTGTGTATTTTATGGGGTTTGTTCATGCGTAAGTTTGTTTATTTAGTTT
TCAATCGTCAATGTGTAGTTCATGATCGCA

**MdMIR156e2**

TACAGACACATATTATTTATAATATTTATAAGAACCTATGATTGCAAAGTACTCCTGCGGCTCTGATAGAT
CTGCTTTTCCAGTACTACTACTACTTCAATCAAACCTGTAAAACATAAGATCCTACAAARGAGAAAAAA
GGGAAATCCAATTATATATACAAGTCAGATCCAAAAATGAGAAATCAGTAAATTTATGAATATACCCAAA
TCACTCGAGACTAAATATGAAATTACAAAGTTAAAACAATATATATGCAAGCAAAGGAAGCATATATATA

TGCAAGCAARGGAAGCATATATAATCGATTAATTAGTAATCAATCAAATAATCATTTTATGAATTAGATTA
ATATGGGAGGTGCAGGGGTGTGGGGGAGGGGCAGGGAAGCTGGATATAGCTAGGTGATGACAGAAGC
ATAGAGAGCACAAAGGAGTGAGATGCATAGTCCATTGCAAGTCATCCATCTGTGCTCTCTATCTTCTGT
CAACAACGTATCACCACTCCCTGCTCTTTCTCTCTCCCAGTTCCCTAGTCCTTACCCTAAATTTAATACT
ATAATATGATTAGAGATTGGAGAGTTGCAGCTATAGCAGATACTTTAAGAGACCTCCACCAATGGTCCTT
ATTTATAGGAGAAAAAGTAGCACCCGCCCACTGAAAAAAGAATGGTAAAAGGTAGATGCAAGTACTC
CTGGGTTTCTCAACTCAGTCACACGGTTACTATAGTACTACGAGTGATTTTTCCAATAGGTCTAAGATAT
TGTTATTTGCAAATTAGTATCACGATTTAATAATATTTTTTTATTTGTAAATTAAAAAATTTTAGATTTGAA
ATTTGTGAAAGATTAGTCGATATCAATTTATAGATCTCATACCTTAGTGTAATATATTATTGTATTAAAAAA
AATTAAAAATACCTTGCAAAGGATAATAAAATTTCTCTTTGTGGCAGAAAAGAAAATAAACACCACCA
CGCAAAGTTATTATTCTCTTTGATTTATAGGACGTATATTTATT

**MdMIR156f1**

GTGGACTCGGCGGATTCCGGTAAGCCGTCGACGGCGGGGAGAGGAAGCTCGATGAAATCGATAAGAG
GGGAAAAGGAGGAGGATCGGAAGCGATTGATGATACTTAAATCCTAAAATGCAAGAGATTATCCAATA
AGCTACCAACTATCATCTTTAAAAATAAAAATACAAATAAAACGTACCGAGCGAAAATTATTTCTAATG
CCAGAATTAAAAATGGACTTATCCAAAATACCTCACCAAACCCACTAAATTTTATTTAATTTTTTAATATC
AATTATTGACATACATGATTTGGGGTGCTTATTGGATAATCTCTTGCATTTTAGGATTTAAGTATCATATCT
TGCTCTGACAAGTCAAACGATAGTGTGTGTGTGTATCATCTTATATGTAGTTGAGAGAGAGTAGTGAGA
GAGAGGGTTAAGGATGGTAGGTGACAGATAGAGAATGAACACATAGAGCTTCTGACATGGATTCATGC
AAGAAAAGAACCACGTGTGCTCACTTTCTTCTGTCACCTTCTTTCCAAACCTGTAATTCTGTATCCGAA
TTATATGTATCCGGTCGTACATGTAGCCACGCATGCATGTTGAACTAAATGAAACACGTACCTTCTTCGT
CCTCTTCTCTTTGTAGCTAGGTTAATTAAGGARGCTGATTTGTGCAAAACAAAGG

**MdMIR156f2**

GGGCACATGAAATTACTCTGGAGTAAGTTCTAACCTTTTCGTACCTTAATTTTCGTATTAATGATWTATT
CGACGAGGTATTAAAAAATACCACAAAATAAARAATTAGTTTAGGCTTTTTAGCCAAAAGRRTCTCCGA
GATTGGCATAAASACAYAAGTGTTAGACGTAAGCAAGAACCGAAGGTTCGACTAGTTGGCCTGGAATC
TCTTGCATTTCAGGACAAGTGATTGGTTGCCGGTTTCCAATAGCATTCAAGGGGAAGAAAATTTAACAT
ATCTTTACCAAGGAGTCAAGGACGGGTCGATGCTTCTGATCCCCTATATAAATTAACCCTCCTAACCCTT
TGTTTTGCACAAATCAGCTTCCTTAATTAACCCTAGCTACAAGAGAAKAGGACGAAGAAGGTACGTGT
TTCATTTAGTTCAACATGCATGCGTGGCTACATGTACGACCGGATACATATAATTCGGATACAGAATTAC
AGGTTTGKAAAGAAGGTGACAGAAGAAAGTGAGCACACGTGGTTCTTTTCTTGCATGAATCCATGTC
AGAAGCTCTATGTGTTCATTCTCTATCTGTCACCTACCATCCTTAACCCTCTCTCTCACTACTCTCTCTCT
CAACTACATATAAGATGATACACACACACACACACTATCGTTTGATTTGTCAGAACAAGATATGATACTT
AAATCCTAAAATGCAAGAGATTATCCAATAAGCACCCCAAATCATGTATGTCAATAATTGTGCACCCCA
AATCATGTATGTCAATAATTGATATTAAAAAACTAAATAAAATTTAGTGGGTTTGGTGAGGTATTTTGGA
TAAGTCCATTTTTAATTCTGGCATTAGAAATAATTTTCGCTCGGTACGTTTTATTTGTATTTTTATTTTTAA
AGATGATCAATTGGTAGCTTCGCCACGACAGTTCATCATTTCGGAACATAGGAAGACTCATAGAGGCAT
TGCAGTCTTTCCTGACCATATAAGCACTAATCTCATAC
TTAGTTTAAT

**2. Predicted secondary structures of the apple precursor miR156s.**

We analyzed RNA secondary structure of the candidate sequences (http://mfold.rna.albany.edu/). The positions of the mature miR156 embedded were marked with highlighted yellow color fonts.

MdMIR156a1

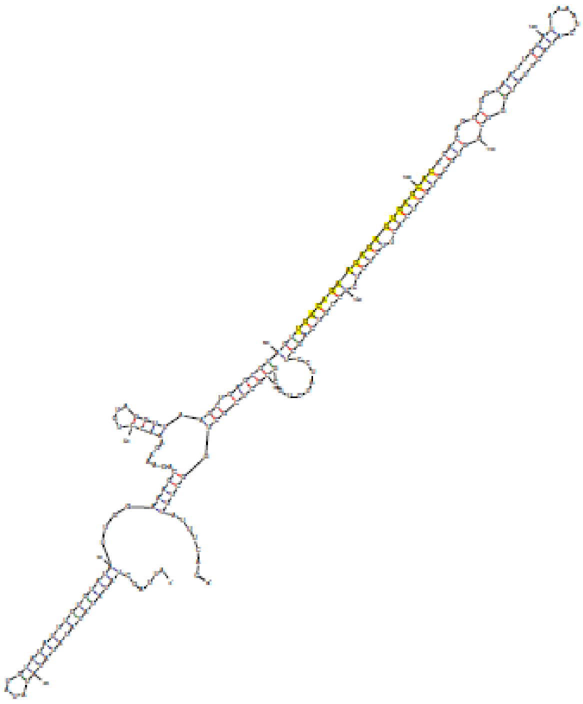

MdMIR156a2

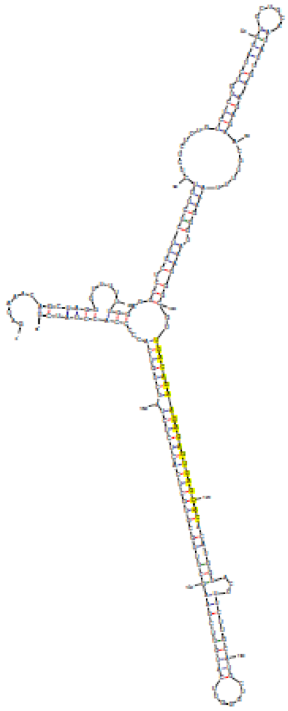

MdMIR156a3

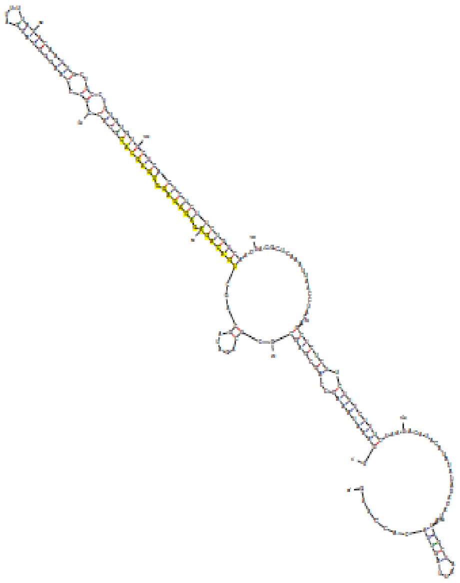

MdMIR156a4

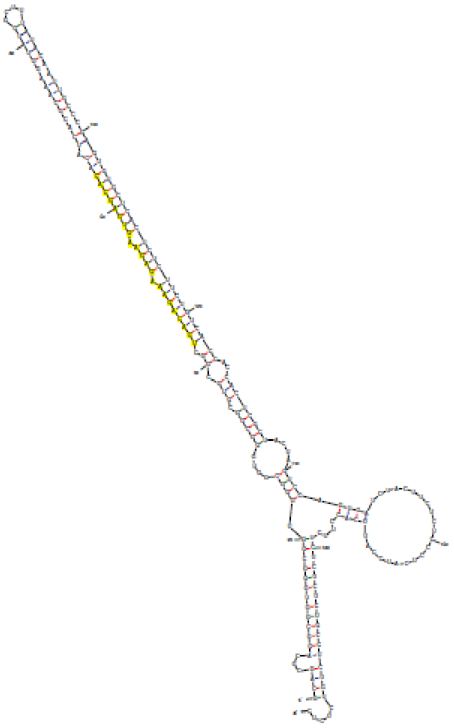

MdMIR156a5

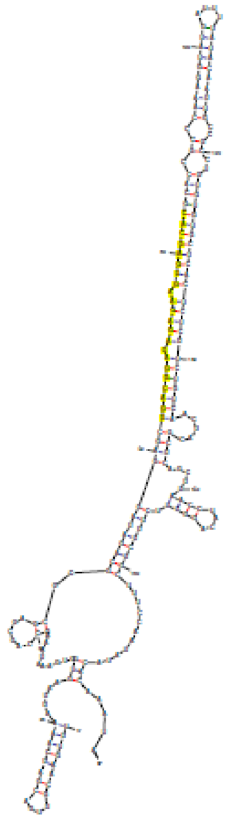

MdMIR156a6

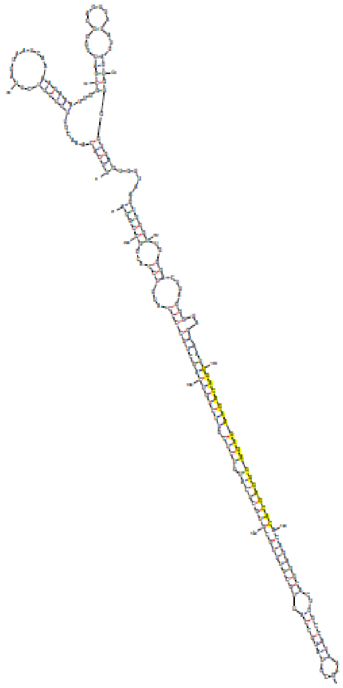

MdMIR156a7

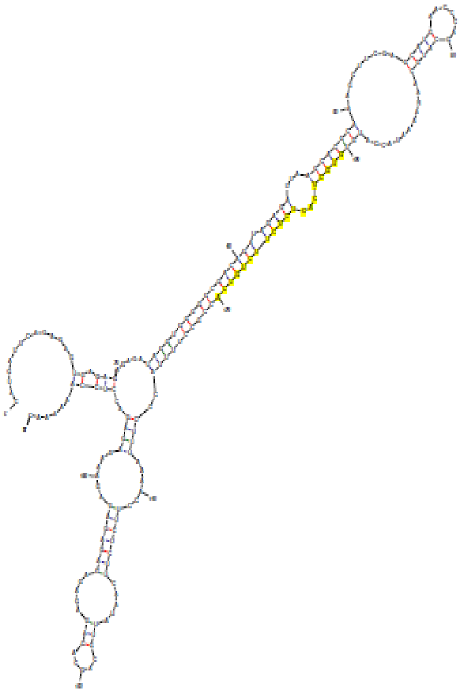

MdMIR156a8

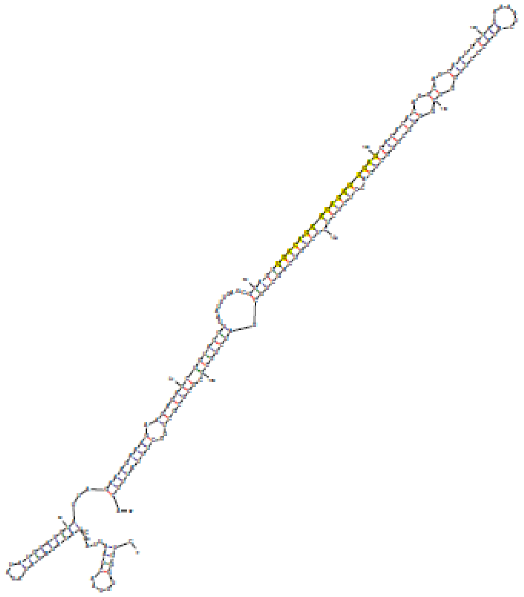

MdMIR156a9

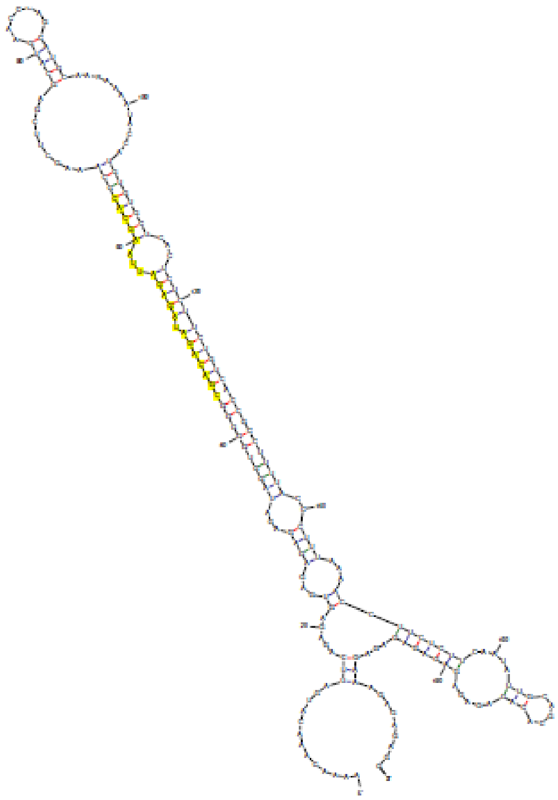

MdMIR156a10

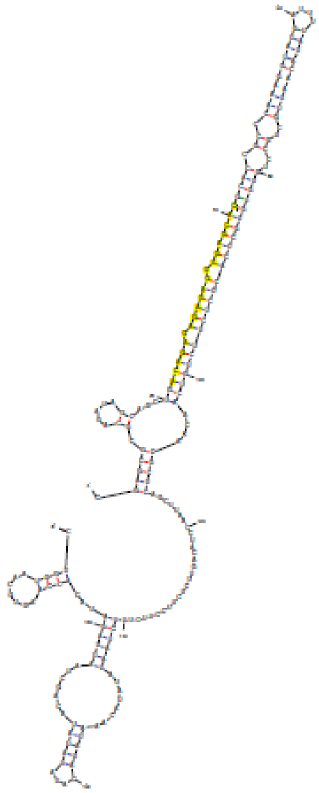

MdMIR156a11

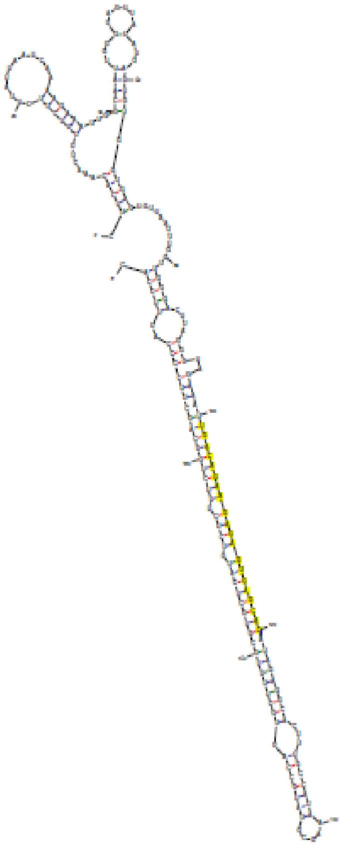

MdMIR156a12

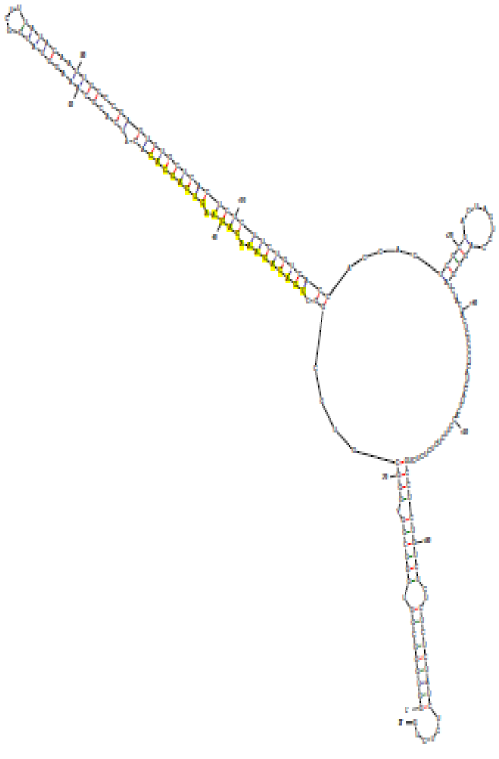

MdMIR156a13

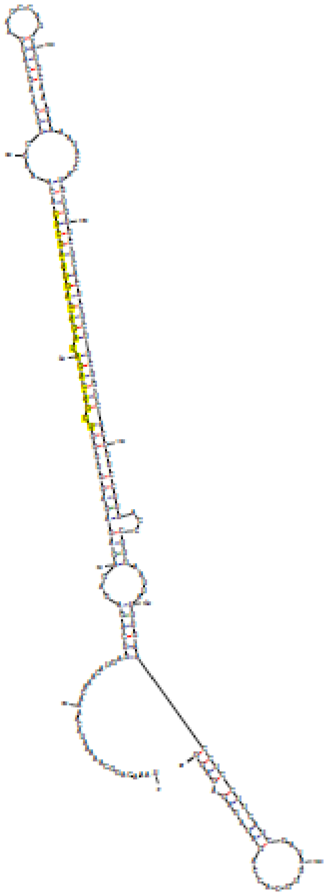

MdMIR156a14

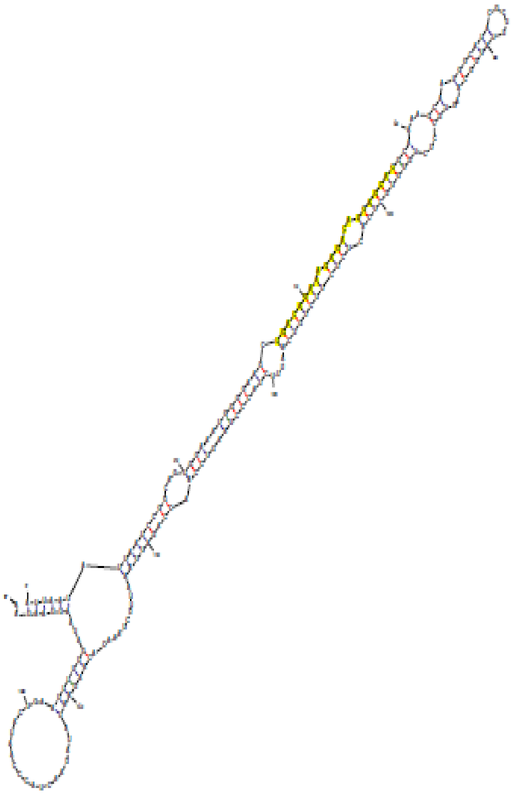

MdMIR156a15

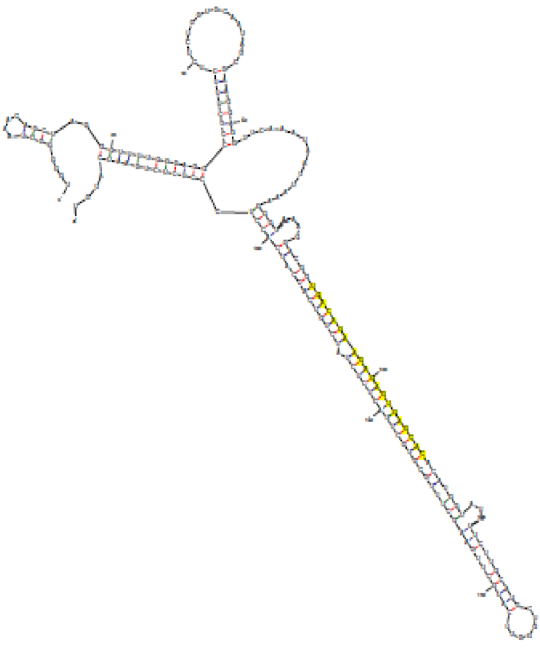

MdMIR156b1

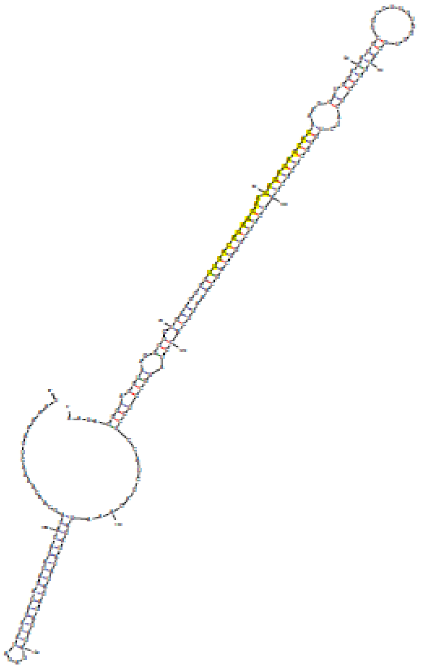

MdMIR156b2

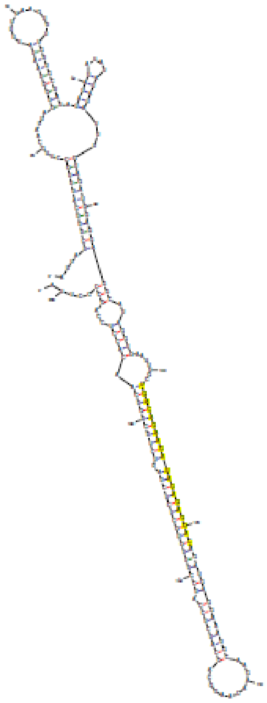

MdMIR156b3

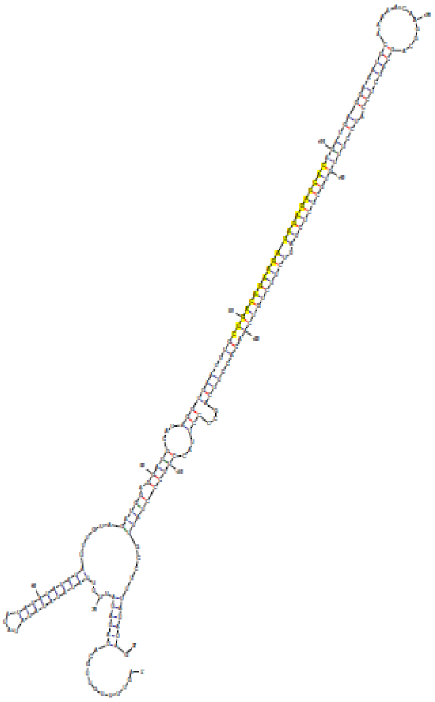

MdMIR156b4

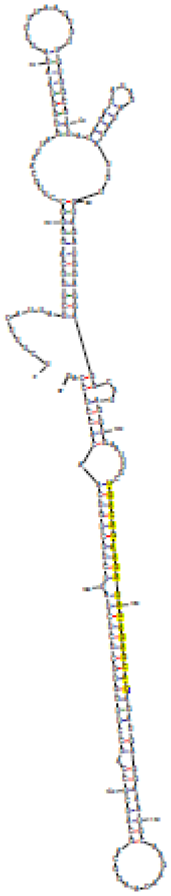

MdMIR156c1

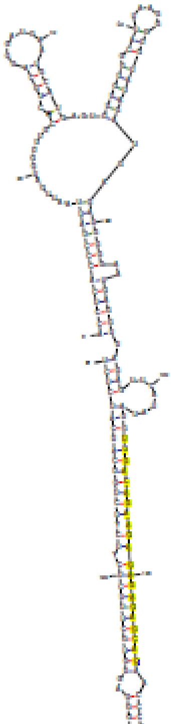

MdMIR156c2

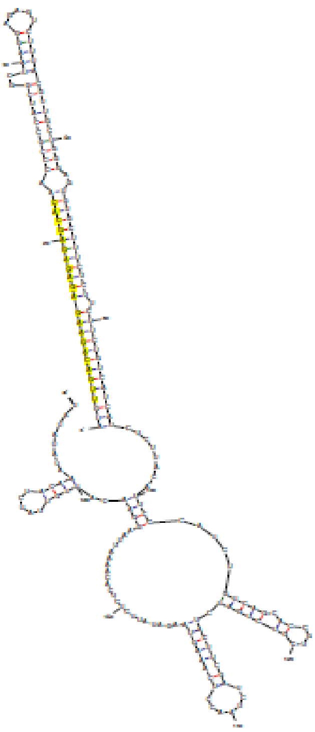

MdMIR156c3

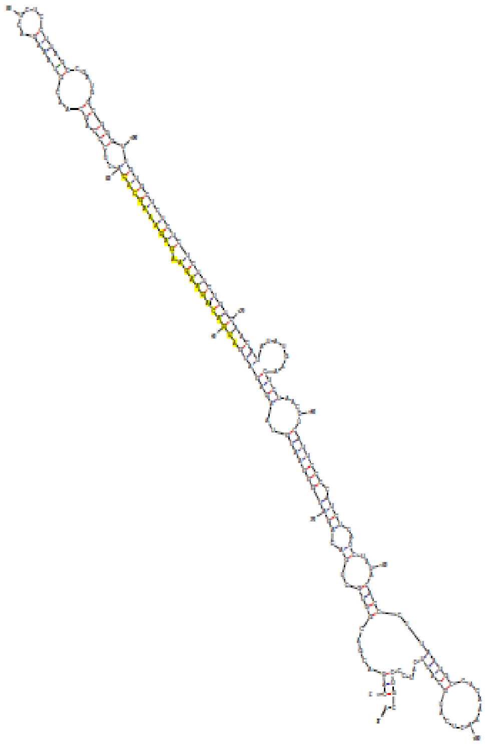

MdMIR156c4

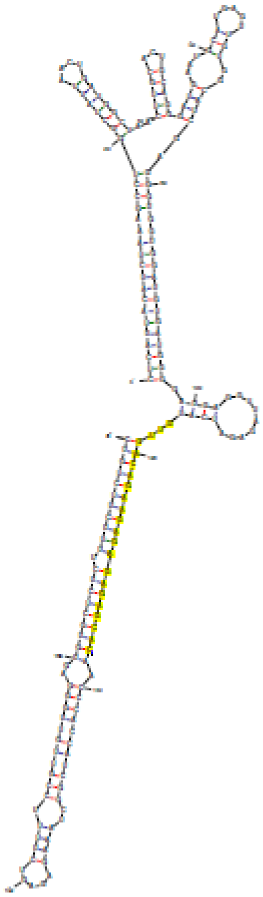

MdMIR156d1

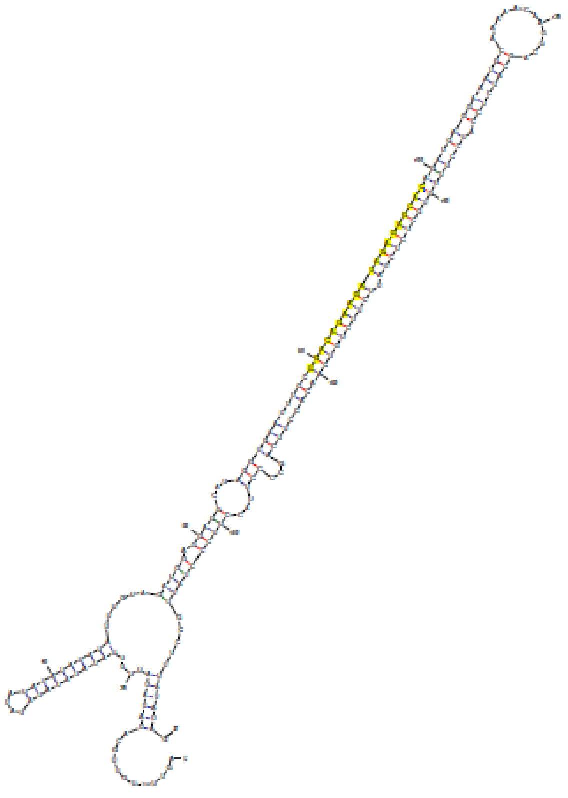

MdMIR156d2

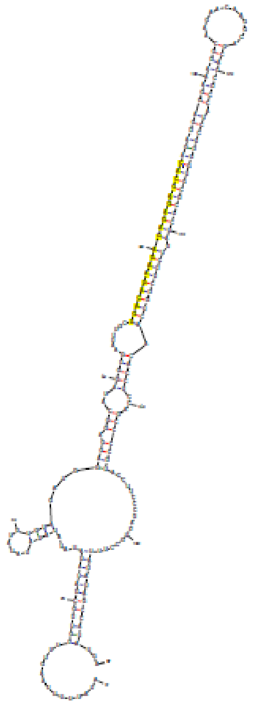

MdMIR156d3

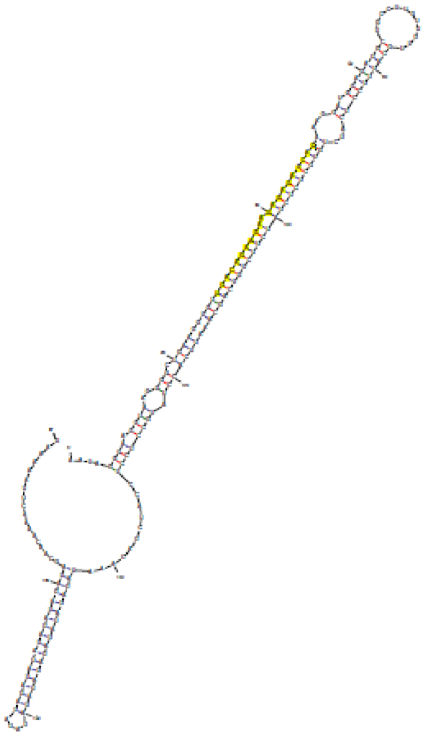

MdMIR156d4

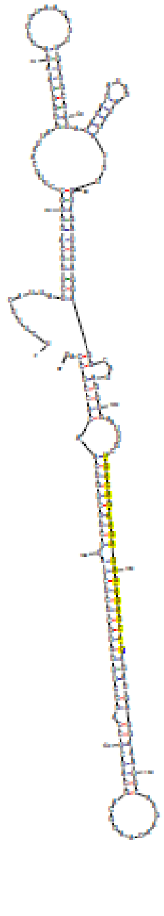

MdMIR156e1

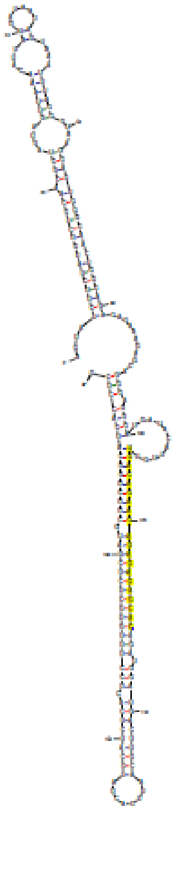

MdMIR156e2

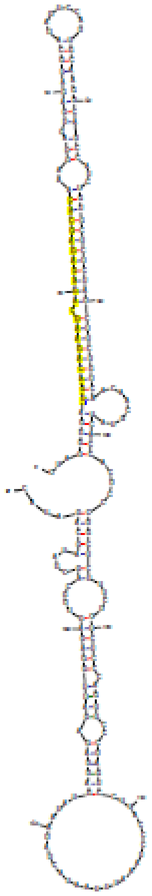

MdMIR156f1

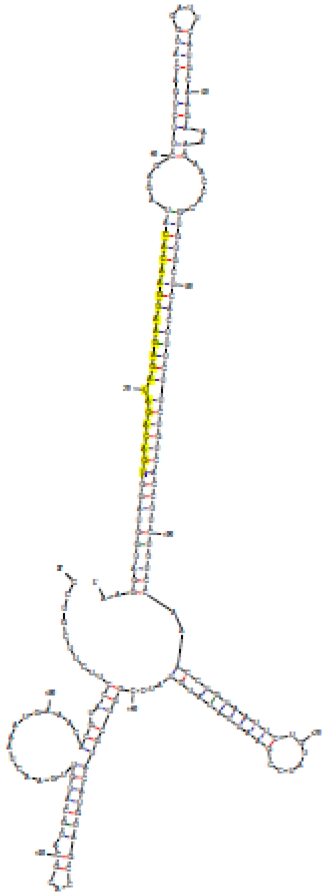

MdMIR156f2

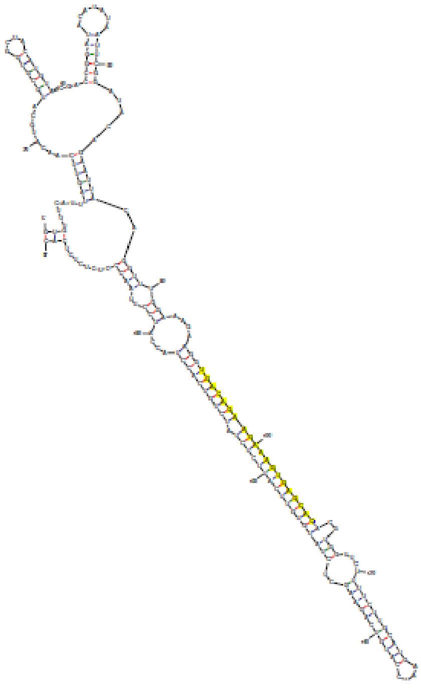

**Table S1 Changes in hydrogen peroxide ($H_2O_2$) concentration in suspension cells of apple 'Orin' leaf cultured in medium supplemented with menadione (MED)**

| Average ±Standard deviation | Days of treatment | | | |
|---|---|---|---|---|
| | 0 | 2 | 4 | 6 |
| Control | 0.21±0.04 | 0.15±0.07cd | 0.19±0.04b | 0.28±0.02c |
| 50 μM MED | 0.21±0.04 | 0.59±0.15a | 0.56±0.02a | 0.54±0.08b |
| 250 μM MED | 0.21±0.04 | 0.32±0.08bc | 0.51±0.16a | 0.41±0.02bc |
| 500 μM MED | 0.21±0.04 | 0.37±0.08b | 0.64±0.22a | 0.80±0.17a |

Three replicates for each treatment. Data values in this table represented as 'average ± standard deviation'. Different letters following data values indicate statistical significance ($p < 0.05$; Duncan's multiple-range test).

**Table S2 Changes in hydrogen peroxide ($H_2O_2$) concentration in suspension cells of apple 'Orin' leaf cultured in medium supplemented with diphenyleneiodonium (DPI)**

| Average ±Standard deviation | Days of treatment | | | |
|---|---|---|---|---|
| | 0 | 2 | 4 | 6 |
| Control | 0.21 ±0.04 | 0.15 ±0.02bc | 0.19 ±0.04b | 0.28 ±0.02c |
| 50 μM DPI | 0.21 ±0.04 | 0.12 ±0.04c | 0.56 ±0.02a | 0.54 ±0.08b |
| 250 μM DPI | 0.21 ±0.04 | 0.33 ±0.12a | 0.51 ±0.16a | 0.41 ±0.02bc |
| 500 μM DPI | 0.21 ±0.04 | 0.30 ±0.08ab | 0.64 ±0.22a | 0.80 ±0.17a |

Three replicates for each treatment. Data values in this table represented as 'average ± standard deviation'. Different letters following data values indicate statistical significance ($p < 0.05$; Duncan's multiple-range test).

**Table S3 Changes in GSH/GSSG ratio in suspension cells of apple 'Orin' leaf cultured in medium supplemented with L-2-oxothiazolidine-4-carboxylic acid (OTC).**

| Average ± Standard deviation | Days of treatment | | | |
|---|---|---|---|---|
| | 0 | 2 | 4 | 6 |
| Control | 2.03 ± 0.11 | 1.70 ± 0.03b | 1.80 ± 0.05b | 2.49 ± 0.04a |
| 25μM OTC | 2.03 ± 0.11 | 1.57 ± 0.29b | 1.79 ± 0.33b | 2.31 ± 0.10a |
| 50μM OTC | 2.03 ± 0.11 | 2.58 ± 0.26a | 2.40 ± 0.27a | 2.34 ± 0.23a |
| 100μM OTC | 2.03 ± 0.11 | 2.93 ± 0.57a | 2.31 ± 0.02a | 2.18 ± 0.30a |

Three replicates for each treatment. Data values in this table represented as 'average ± standard deviation'. Different letters following data values indicate statistical significance ($p < 0.05$; Duncan's multiple-range test).

**Table S4 Changes in GSH/GSSG ratio in suspension cells of apple 'Orin' leaf cultured in medium supplemented with Buthionine Sulphoximine (BSO).**

| Average ±Standard deviation | Days of treatment | | | |
|---|---|---|---|---|
| | 0 | 2 | 4 | 6 |
| Control | 2.03±0.11 | 1.70±0.03a | 1.80±0.05a | 2.49±0.04a |
| 0.1mM BSO | 2.03±0.11 | 0.70±0.18bc | 1.37±0.05b | 1.55±0.25b |
| 0.5mM BSO | 2.03±0.11 | 0.93±0.33b | 1.25±0.16b | 0.87±0.09c |
| 1mM BSO | 2.03±0.11 | 0.47±0.26c | 0.47±0.17c | 0.36±0.07d |

Three replicates for each treatment. Data values in this table represented as 'average ± standard deviation'. Different letters following data values indicate statistical significance ($p < 0.05$; Duncan's multiple-range test).

**Table S5 Primer sequences for reverse transcription and RT-PCR of mature miR156 and 5s RNA**

| Primers | Sequence (5′→3′) |
|---|---|
| RT-miR156 | GTCACATCGTATCGTGAAGCTGCGCAGCTGATGTGACGTGCTCAC |
| miR156 | F: TGCACTAGCGTGTGACAGAAGA |
| | R: ACATCGTATCGTGAAG |
| RT-5s rRNA | GTCACATCGTATCGTGAAGCTGCGCAGCTGATGTGACTGGATTGG |
| 5s rRNA | F: TGCACTAGCGTGTAGAGGAACC |
| | R: ACATCGTATCGTGAAG |

**Table S6 Primer sequences of *MdMIR156s MdHYL1* and *MdSPLs***

| Primers | Sequence (5′→3′) |
|---|---|
| *MdMIR156 a1* | F:GCTGGGAGTAAGAGGGTATCTA |
| | R:CTCCTTCTGCTCTCCCAATTC |
| *MdMIR156 a5* | F:AGCTGACGGAGAGAGAAGT |
| | R:CTGAGCGTAGTTGACAGAAGAG |
| *MdMIR156 a6* | F: ACAGAGGCACTTGCCATAAA |
| | R: TAATCCGGCAGTGGAAATGG |
| *MdMIR156 a9* | F:AGCTGACGGAGAGAGAAGT |
| | R:GAGAGTGAGCACACATGGTATT |
| *MdMIR156 a10* | F:CAGGAAGGAGAATTGGGAGAAA |
| | R:TCTCTTCCCGCAAAGGAATG |
| *MdMIR156 a12* | F:CACGCAAAGGTATGGCTTTATAC |
| | R:GCAGAGAGAGAGAAGGTGAATG |
| *MdMIR156 d1* | F:GACAGAAGATAGAGAGCACAGATG |
| | R: AGGTGTTGACAGAAGAAT |
| *MdHYL1* | F: TTCCCATGCGCCTTCTTT |
| | R: GCTGACTGCTCTGCTATCTTAC |
| *MdSPL3* | F: CTCATCAGCAGTCGGCGTCA |
| | R:TCGGGGTTAAGAGAAAGGGCAG |
| *MdSPL4* | F:CCCATGTACCTTCATGGAAAGTCAAG |
| | R:GCAAATGTTTGCCCACCATTTCT |
| *MdSPL7* | F:TGGTGGACTTTGATAAGGTGAAGAGA |
| | R:ACTTCGTTCGTCAGTTGCGGA |
| *MdSPL10&11* | F:GGAGCATGAGAGAAACTTTTTTCAAGTA |
| | R:CACATTGTTGCTTGCACGCC |
| *MdSPL12* | F:GTCCTTGCCATGCCCGAT |
| | R:TCAGGTTTTCCAGTTCCCACA |
| *MdSPL18* | F:AGGTTCAGCAGGCGGGACTA |
| | R:TCGTCGGAAAGTGATTGGAGG |
| *MdSPL20* | F:GCCAAGAGATACTCCCTACTGACATTC |
| | R:CTGGAACTGCTGAAGCTGTGTTG |
| *MdSPL21&22* | F:CAAGATATTCGTGCGGATTCGATTAA |
| | R:CTGTGGAAATGTTGGAATTCTGATGC |
| *MdSPL23* | F:TCCCCTGATGTCCGAGTGCT |
| | R:TTTCAGATGCGTGCCCCGAC |
| *MdSPL24* | F:GCTTACCGAGAAAAACAAATCCCAT |
| | R:AGACTGAACTGGACTGTGCTGTGT |
| *MdSPL26* | F:GTCACCACTACTCATCCAGGTCAGA |
| | R:TCCTCCTCATCATGTGAGCATGAGAATT |
| *β-Actin* | F:TGGTGAGGCTCTATTCCAAC |
| | R:TGGCATATACTCTGGAGGCT |

**Table S7 Detailed list of MIR156 gene family in apple**

| MiRNA name | Sequence | Contig | Length | Strand | Match position | Star sequence |
|---|---|---|---|---|---|---|
| MIR156 a1 | TGACAGAAGAGAGTGAGCAC | MDC010428.317 | 20 | + | 6232 | TGACAGAAGAGAGTGAGCAC |
| MIR156 a2 | TGACAGAAGAGAGTGAGCAC | MDC015254.139 | 20 | + | 2152 | ACTGTCTATCTCTCACTCGTG |
| MIR156 a3 | TGACAGAAGAGAGTGAGCAC | MDC010428.318 | 20 | - | 7697 | GTGCTCACTCTCTTCTGTCA |
| MIR156 a4 | TGACAGAAGAGAGTGAGCAC | MDC007399.176 | 20 | - | 6603 | GTGCTCACTCTCTTCTGTCA |
| MIR156 a5 | TGACAGAAGAGAGTGAGCAC | MDC010428.318 | 20 | - | 17326 | GTGCTCACTCTCTTCTGTCA |
| MIR156 a6 | TGACAGAAGAGAGTGAGCAC | MDC018927.245 | 20 | + | 11206 | ACTGTCTTTCTCTGTACTCGTG |
| MIR156 a7 | TGACAGAAGAGAGTGAGCAC | MDC022546.57 | 20 | - | 2800 | CACGAATTAGAGATAGACAGC |
| MIR156 a8 | TGACAGAAGAGAGTGAGCAC | MDC010428.308 | 20 | + | 6933 | ACTGTCTCTCTCTCCACTC |
| MIR156 a9 | TGACAGAAGAGAGTGAGCAC | MDC002074.373 | 20 | - | 2158 | ACTGTCTATCTCTCACTCGTG |
| MIR156 a10 | TGACAGAAGAGAGTGAGCAC | MDC038184.5 | 20 | - | 20083 | GTGCTCACTCTCTTCTGTCA |
| MIR156 a11 | TGACAGAAGAGAGTGAGCAC | MDC022169.138 | 20 | + | 7417 | ACTGTCTTTCTCTGTACTCGTG |
| MIR156 a12 | TGACAGAAGAGAGTGAGCAC | MDC015520.219 | 20 | - | 7939 | GTGCTCACTCTCTTCTGTCA |
| MIR156 a13 | TGACAGAAGAGAGTGAGCAC | MDC013580.195 | 20 | - | 17918 | GTGCTCACTCTCTTCTGTCA |
| MIR156 a14 | TGACAGAAGAGAGTGAGCAC | MDC022241.434 | 20 | - | 30246 | GTGCTCACTCTCTTCTGTCA |
| MIR156 a15 | TGACAGAAGAGAGTGAGCAC | MDC011231.822 | 20 | + | 32339 | ACTGTCTATCTCTCATTCGTG |
| MIR156 b1 | CTGACAGAAGATAGAGAGCAC | MDC007657.477 | 21 | - | 1890 | GTGCTCACTCTCTTCTGTCA |
| MIR156 b2 | CTGACAGAAGATAGAGAGCAC | MDC018442.71 | 21 | + | 5569 | ACTGTCTTCTTATCTCTCGTG |
| MIR156 b3 | CTGACAGAAGATAGAGAGCAC | MDC012217.482 | 21 | + | 9927 | AACTGTCTTCTTATCTCTCGTG |
| MIR156 b4 | CTGACAGAAGATAGAGAGCAC | MDC018690.154 | 21 | + | 10957 | ACTGTCTTCTTATCTCTCGTG |
| MIR156 c1 | TTGACAGAAGAGAGAGAGCAC | MDC038783.9 | 21 | + | 1407 | TACTGTCTYCTYCTCTTTCGTG |
| MIR156 c2 | TTGACAGAAGAGAGAGAGCAC | MDC008280.307 | 21 | + | 3 | ACTGTCTTCTTCTCTTTCGTG |
| MIR156 c3 | TTGACAGAAGAGAGAGAGCAC | MDC019519.287 | 21 | - | 801 | GTGCTCTCTCTCTTCTGTCAA |
| MIR156 c4 | TTGACAGAAGAGAGAGAGCAC | MDC019519.284 | 21 | + | 5666 | CTGTCTTCTTTTCTCTCGTG |
| MIR156 d1 | TGACAGAAGATAGAGAGCAC | MDC012217.482 | 20 | + | 9928 | ACTGTCTTCTTATCTCTCGTG |
| MIR156 d2 | TGACAGAAGATAGAGAGCAC | MDC018442.71 | 20 | + | 5570 | ACTGTCTTCTTATCTCTCGTG |
| MIR156 d3 | TGACAGAAGATAGAGAGCAC | MDC007657.477 | 20 | - | 1890 | GTGCTCTCTATCTTCTGTCA |
| MIR156 d4 | TGACAGAAGATAGAGAGCAC | MDC018690.154 | 20 | + | 10958 | ACTGTCTTCTTATCTCTCGTG |
| MIR156 e1 | TTGACAGAAGATAGAGAGCAC | MDC002621.233 | 21 | + | 15660 | ACTGTCTTCGTATCTCTCGTG |
| MIR156 e2 | TTGACAGAAGATAGAGAGCAC | MDC002441.399 | 21 | - | 5519 | GTGCTCACTCTCTTCTGTCA |
| MIR156 f1 | TGACAGAAGAAAGTGAGCAC | MDC013721.403 | 20 | - | 6675 | CACAAGTAAGAGATAGACAGT |
| MIR156 f2 | TGACAGAAGAAAGTGAGCAC | MDC013721.399 | 20 | + | 9390 | ACTGTCTATCTCTTACTTGTG |

**Table S8 Primer sequences of *MdRboh* family members**

| Primers | Sequence (5′→3′) |
|---|---|
| *Rboh D1* | F:GCACCAAGTTACCACACAAATC |
| MDP0000703059 | R:GTTCCCGTGTCACCCAATAA |
| *Rboh D2* | F:GCAGAGAGTCTGGGTTATGATG |
| MDP0000262620 | R:CTCGAATGCCTCCTCTCTTTC |
| *Rboh D3* | F:CGGCACAGGACTACAAGAAATA |
| MDP0000421679 | R:TCATCTTCCTTGCCCTTCATC |
| *Rboh H1* | F:ATGGGCATCAAGAGACATCTG |
| MDP0000160005 | R:TGCTCCCTTGTTACCCAATAAA |
| *Rboh H2* | F:GATGCGAGGTCTGCTCTTATT |
| MDP0000195681 | R:TGAGTTCTTATCCGGCTTTCTG |
| *Rboh K1* | F:CTAATAAGCACTATACAAGCGTTGC |
| MDP0000280452 | R:TGAGAAGATGTTGAACCAGTCC |
| *Rboh K2* | F:TTTGGTGACCCTTCAGTCT |
| MDP0000261507 | R:CGGAGTCCGGGAGAGAA |

**Table S9 Primer sequences of *MdHXK* family members**

| Primers | Sequence (5′→3′) |
|---|---|
| *HXK1* | F: CTTTCTGCTGCTGGGATCTT |
| MDP0000181206 | R: TGGTGCGGAATTCAGTGTAG |
| *HXK2* | F: GCAATGGATGGTGGTTTGTATG |
| MDP0000309677 | R: GTTTGAGTGCTCGACAGAAATG |
| *HXK3* | F: GGTAGCTTTGGATGGTGGATTA |
| MDP0000823956 | R: ATCCCAGACCCATCGTTAGA |
| *HXK4* | F: GGAACGTGCACATGCAATAC |
| MDP0000643891 | R: CTGTGAAGCAGTGAAGGAGTT |
| *HXK5* | F: CGGAGAGGAAGTTGCAGAAA |
| MDP0000849870 | R: CGATTCCTAGGTATTGTGAGTGAG |
| *HXK7* | F: GAGCAAGATATTGGGACGATGA |
| MDP0000251415 | R: GGCCCTGTAGTTTGGGAATAG |
| *HXK8* | F: CACTAGATCTTGGCGGTACTAAC |
| MDP0000175245 | R: GAGGAATGGCTTGTCGATACA |
| *HXK9* | F: CCTGGAGCTAAGCTGTGAAA |
| MDP0000316781 | R: CCATCGGGTAGTTTATCCACAA |
